# Supplementary material for: The Arabidopsis F-box protein FBW2 targets AGO1 for degradation to prevent spurious loading of illegitimate small RNA
Source: Cell Rep. 2022 Apr 13;39(2):110671. doi: 10.1016/j.celrep.2022.110671 (PMC9035678; doi:10.1016/j.celrep.2022.110671)
Supplement: Document S1. Figures S1–S9 [file mmc1.pdf]

Cell Reports, Volume *39*

Supplemental information

The Arabidopsis F-box protein FBW2 targets AGO1 for degradation to prevent spurious loading of illegitimate small RNA

Thibaut Hacquard, Marion Clavel, Patricia Baldrich, Esther Lechner, Imma Pérez-Salamó, Mikhail Schepetilnikov, Benoît Derrien, Marieke Dubois, Philippe Hammann, Lauriane Kuhn, Danaé Brun, Nathalie Bouteiller, Nicolas Baumberger, Hervé Vaucheret, Blake C. Meyers, and Pascal Genschik

Supplemental information

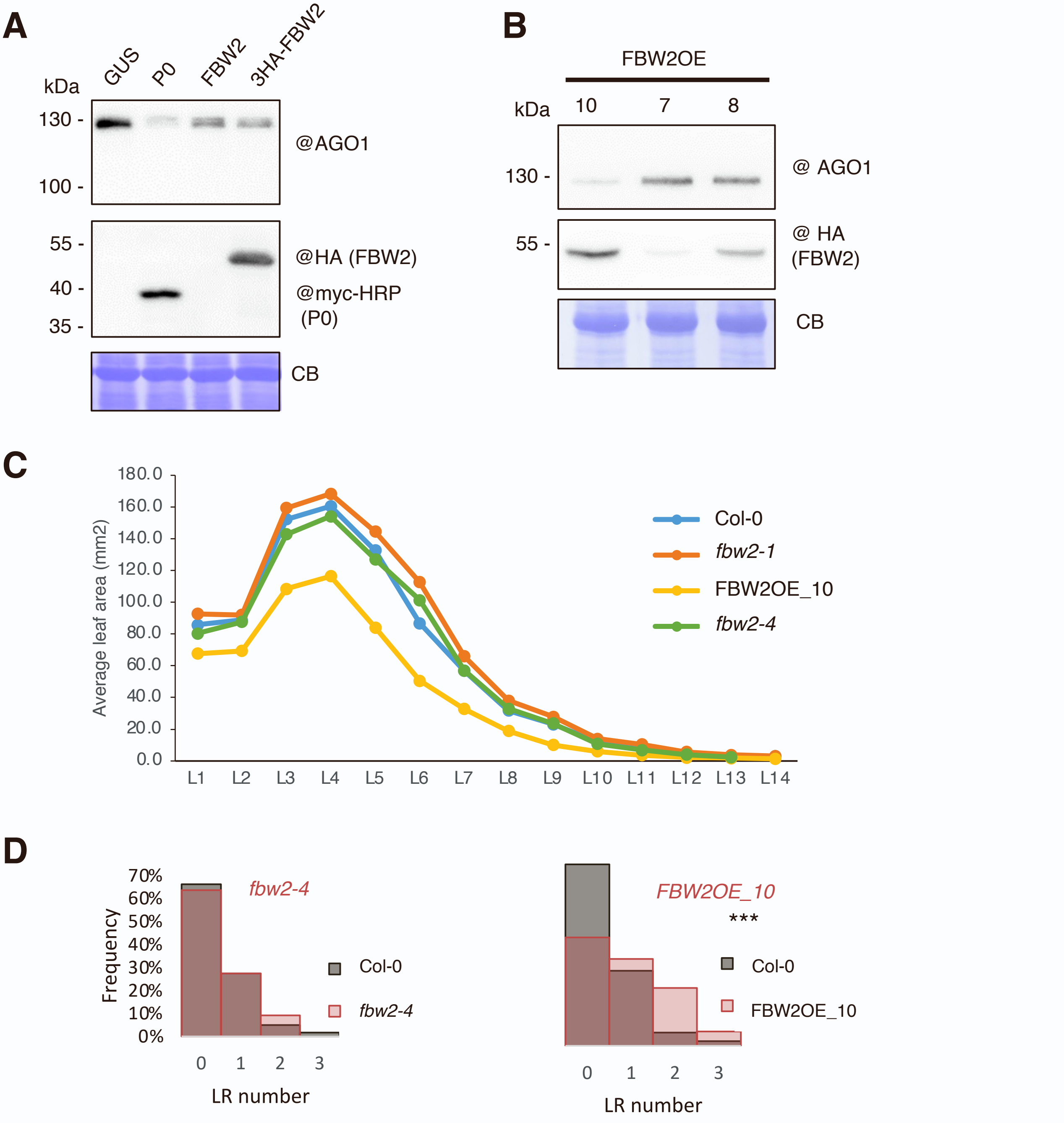

Figure S1. Overexpression of 3HA-FBW2 *in planta* and phenotypic characterization of the mutants (related to Figure 1). (**A**) Western blot of protein extracts from 4 week-old *N. benthamiana* agro-infiltrated leaves. Agrobacteria harbouring binary vectors for the expression of 35S:CFP-AGO1 combined with either 35S:GUS (control), 35S:P0-6myc, 35S:FBW2 and 35S:3HA-FBW2 constructs were infiltrated at an OD of 0,3 and tissues were sampled 3 days later. Coomassie blue (CB) staining was used as loading control. The "@" symbol indicates hybridization with the corresponding antibody.
(**B**) Three representative independent homozygous, simple insertion, FBW2OE (35S:3HA-FBW2) transgenic lines expressing variable amount of 3HA-FBW2 protein. Protein extracts from 7 day-old seedlings grown on MS medium were analysed by western blot. Coomassie blue (CB) staining was used as loading control. The "@" symbol indicates hybridization with the corresponding antibodies.
(**C**) Average area of each individual leaf, from old to young (L1-L14), of *fbw2-1*, *fbw2-4* and the FBW2OE line 10. Measurements were performed at 22 days after stratification of plants grown in soil. Ten plants were measured for each line.
(**D**) Number of lateral roots of young seedlings of *fbw2-4* and FBW2OE line 10. Lateral roots were counted at 8 days after stratification after growth *in vitro* on vertical petri dishes. Lateral roots were counted under a binocular (lateral root stage >VIII) for at least 10 plants per line. *** = P<0.001, t-test.

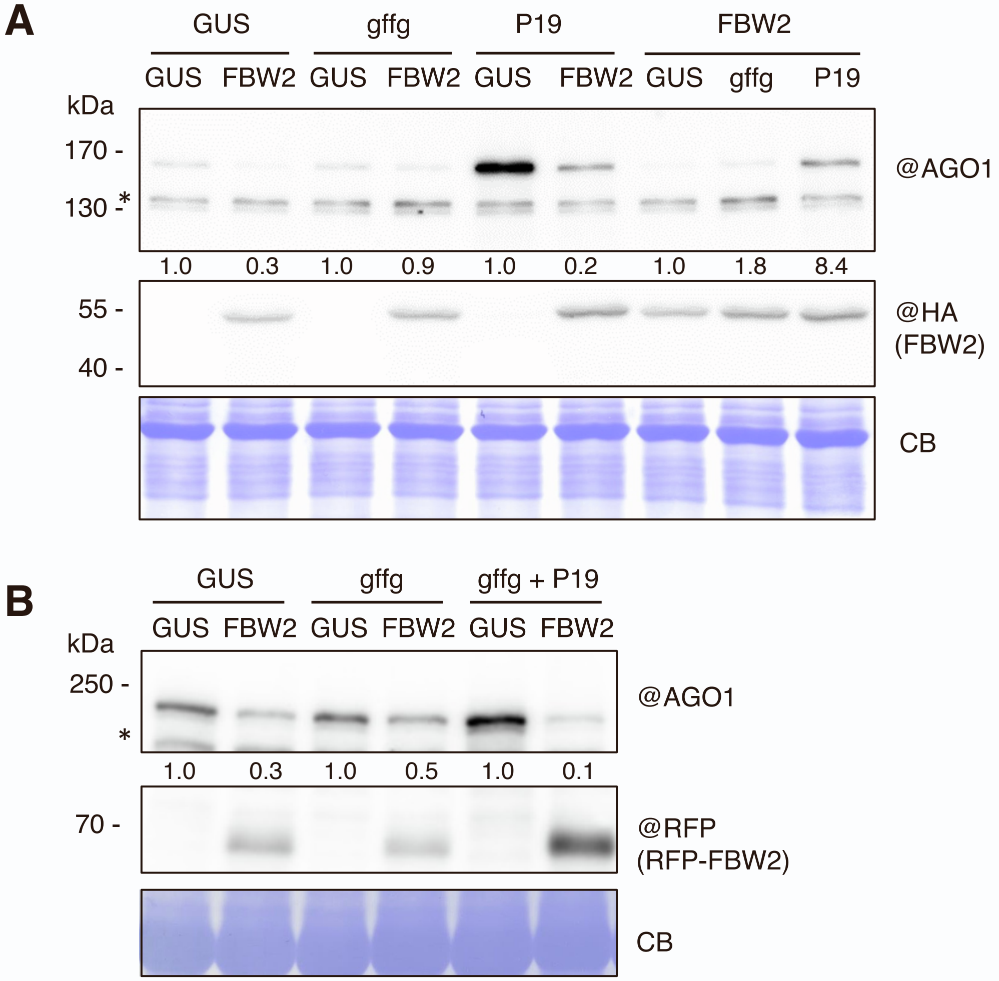

Figure S2. Effect of the inverted repeat *gffg* and P19 overexpression on AGO1 degradation by FBW2 (related to Figure 1).
(**A**) Western blot of protein extracts from 4 week-old *N. benthamiana* agroinfiltrated leaves with Agrobacteria harbouring the following binary vectors: 35S:CFP-AGO1, 35S:GUS (control), 35S:3HA-FWB2, 35S:gffg and 35S:P19 in combinations as specified. All constructs were infiltrated at 0,3 OD and tissues for protein analysis were sampled 3 days later. Coomassie blue (CB) staining was used as loading control. For this panel and also for panel B, AGO1 signal was quantified by ImageJ, normalized to the corresponding CB. Numbers are indicated below the panel as relative to the control set at 1.0. The "*" symbol indicates an aspecific cross-reacting band. The "@" symbol indicates hybridization with the corresponding antibodies.
(**B**) Western blot of protein extracts from 4 week-old *N. benthamiana* agroinfiltrated leaves with Agrobacteria harbouring the following binary vectors: 35S:RFP-AGO1, 35S:GUS (control), 35S:RFP-FWB2, 35S:gffg and 35S:P19 in combinations as specified. All constructs were infiltrated at 0,2 OD except for the experiment with P19 for which the infiltration solutions were adjusted to compensate the difference of expression (0.05 for AGO1, 0.1 for GUS and 0.1 for FBW2). Coomassie blue (CB) staining was used as loading control. The "*" symbol indicates an aspecific cross-reacting band. The "@" symbol indicates hybridization with the corresponding antibodies.

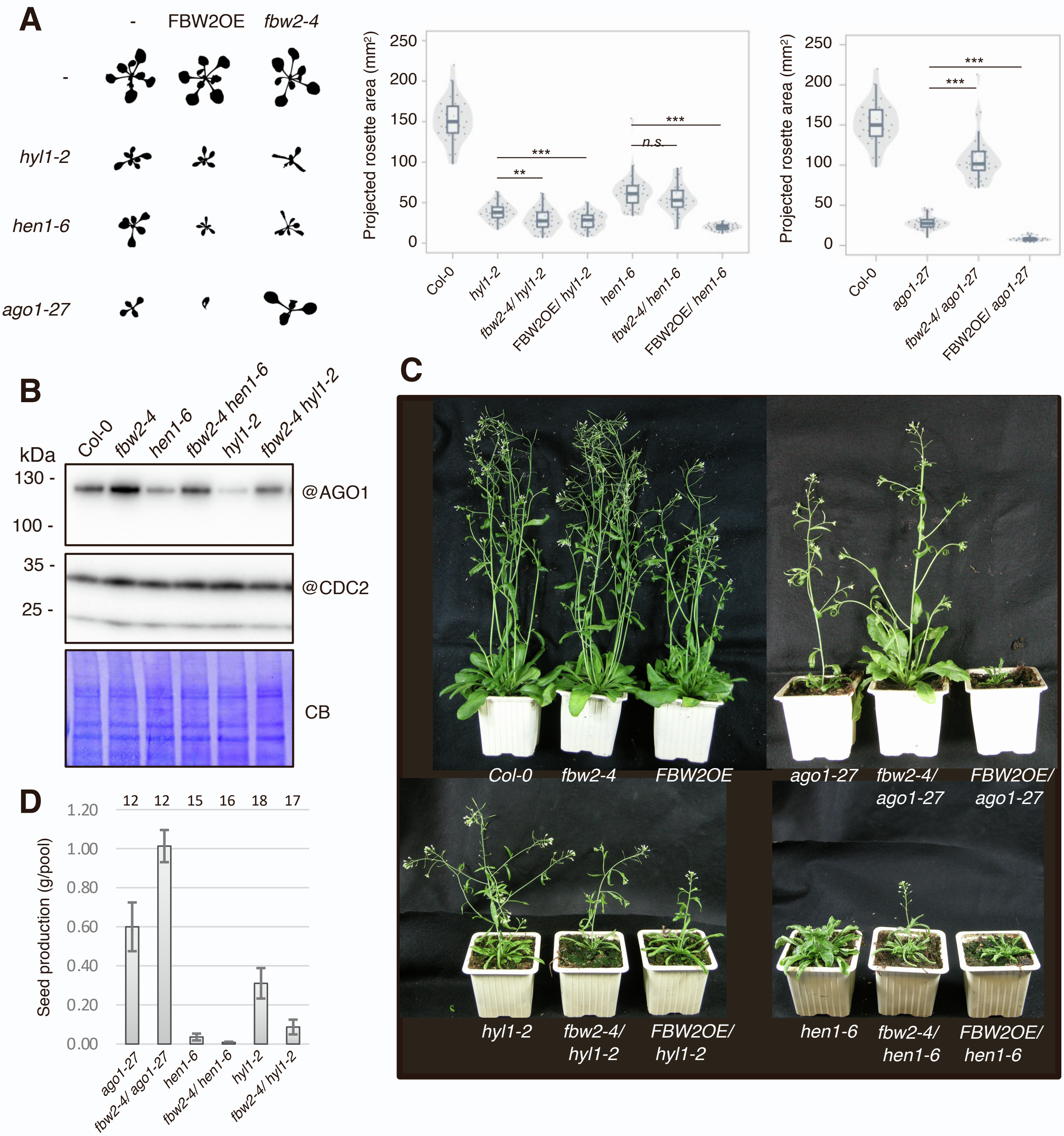

Figure S3. AGO1 protein level and phenotype of *hen1-6 fbw2-4* and *hyl1-2 fbw2-4* double mutants (related to Figure 4).
(**A**) Shape imprints and measurements of the rosette area of 17-days-old *in vitro* grown seedlings. Individual values are represented as dots within violin plots. Three independent biological replicates are shown, with n~12 plants per repeat. Statistical significance is shown only for the comparisons of the crosses with the single *hyl1-2, hen1-6* or *ago1-27* mutant. *** P < 0.001 (Students t-test), ** P < 0.01 (Students t-test), n.s. = not significant (P > 0.05, Students t-test).
(**B**) Western blot of protein extracts from 5 day-old seedlings of the indicated genotypes. Hybridization with the CDC2 antibody and Coomassie blue (CB) staining were used as loading controls.
(**C**) Pictures of three representative 40 days-old plants of Col-0, *hyl1-2*, *hen1-6*, *ago1-27* and their crosses with *fbw2-4* or 35S:3HA-FBW2 (FBW2OE) as indicated.
(D) Measurements of seed production (in gram). For each genotype, seeds from two individual plants were pooled and subsequently weighted. The number of pools measured per genotype is indicated on top of the graphs.

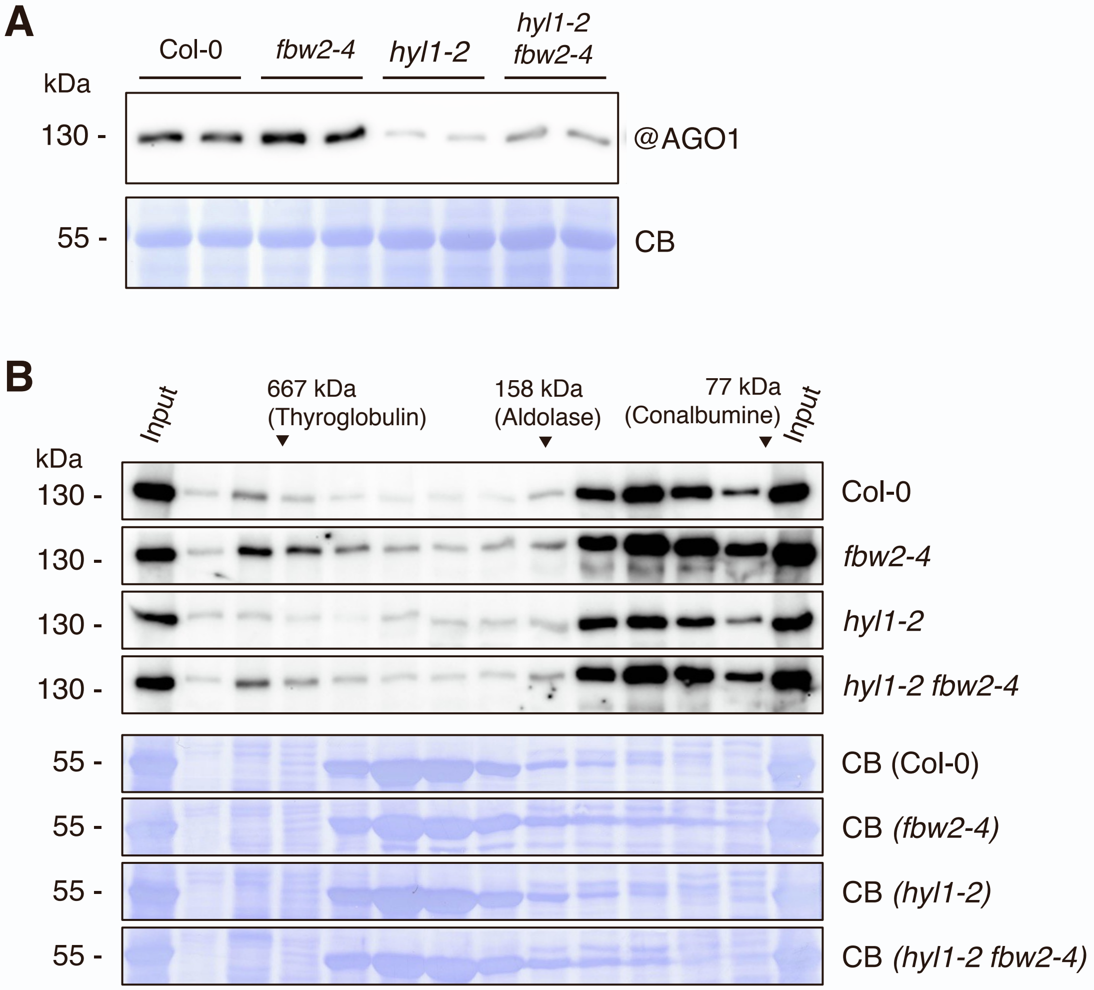

Figure S4. Loss of *FBW2* restores high molecular weight AGO1 complexes in *hyl1-2* (related to Figure 4).
(**A**) Western blot of protein extracts of 13 day-old seedlings of Col-0, *fbw2-4*, *hyl1-2*, and *hy1-2 fbw2-4*. Two biological replicates were made to illustrate the tendency of AGO1 homeostasis.
(**B**) Gel filtration analysis of AGO1-based RISC complexes from the same protein extracts shown in (A). Western blots of protein extracts from eluates of the gel filtration column (superdex 200 10/300 increase column on an AKTA Pure system) spanning from 6.75 to 13.75 ml elution volume. Molecular weights (kDa) of known protein sizes are indicated on top of the blot. "@" indicates hybridization with the corresponding antibodies. Coomassie blue staining was used as loading control.

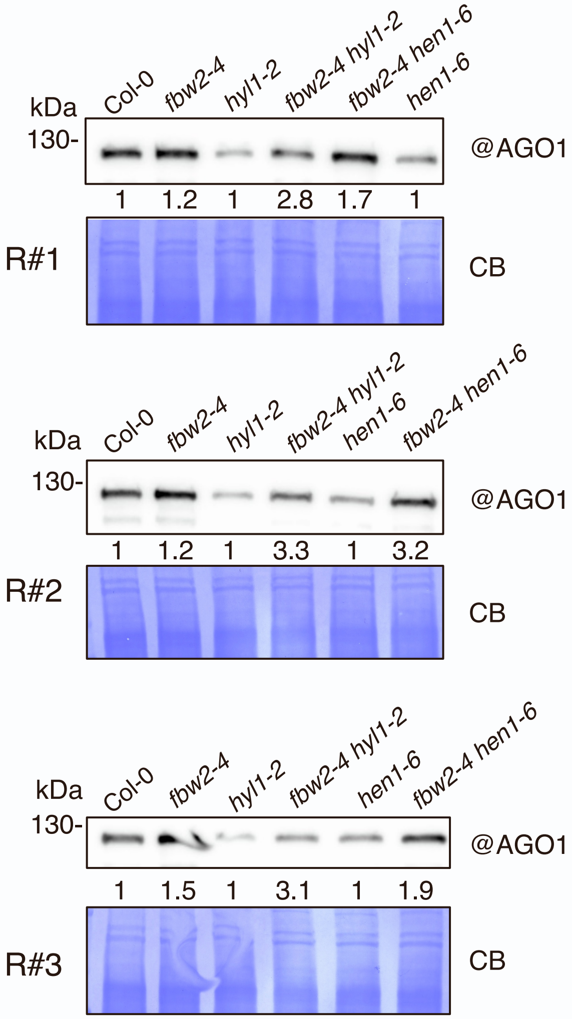

Figure S5. AGO1 protein level in different mutants used for RNA deep-sequencing experiments (related to Figure 5). Western blot of protein extracts from 8 day-old seedlings of the indicated genotypes. R#1, R#2, R#3 correspond to the three biological replicates. Hybridization with the @AGO1 antibody. Coomassie blue (CB) staining were used as loading controls. AGO1 signal was quantified by ImageJ, normalized to the corresponding Coomassie blue signal. Numbers are indicated below the panel as relative to Col-0, *hyl1-2 and hen1-6* set at 1.0.

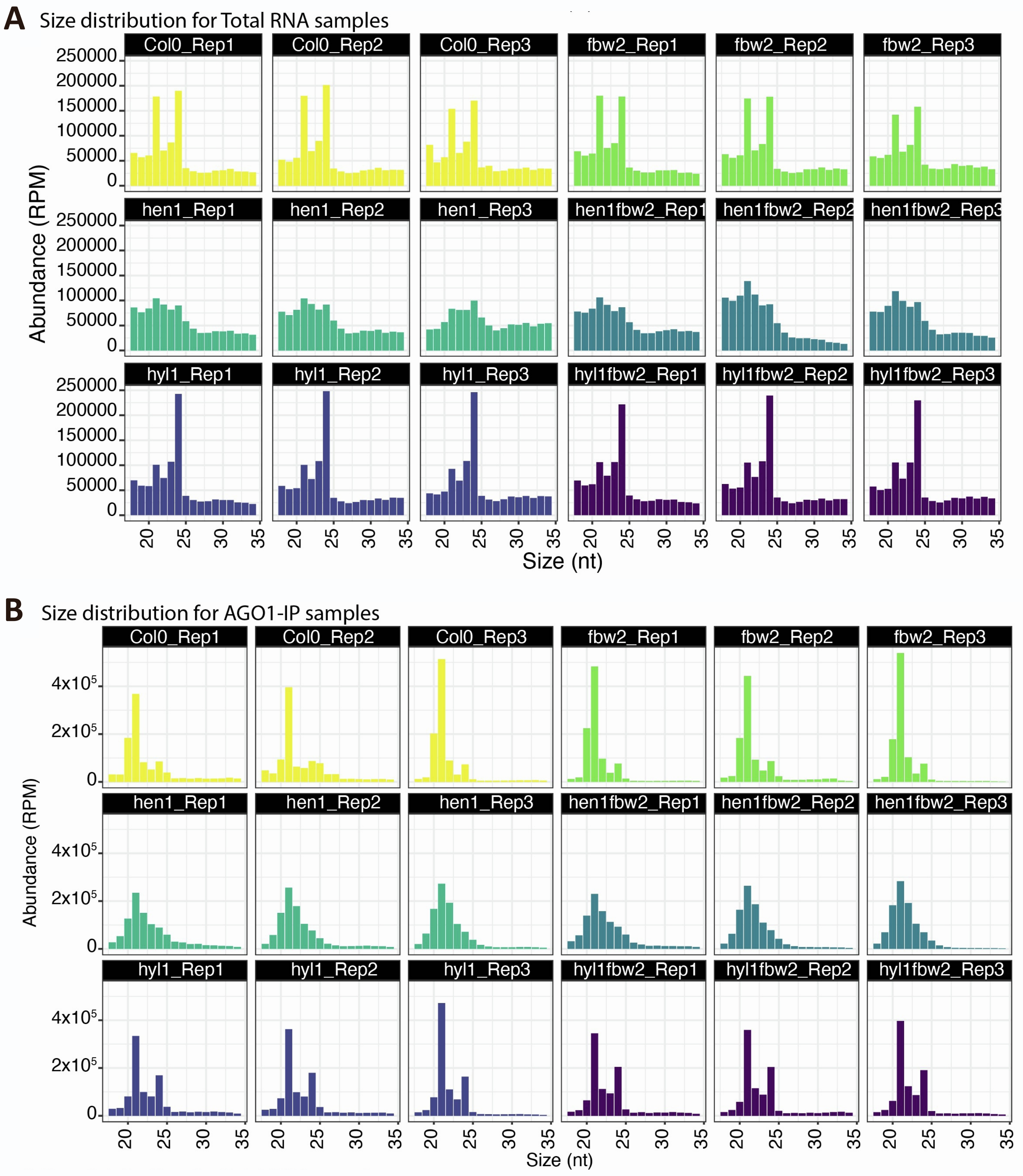

Figure S6. *FBW2* mutation has no effect in small RNA size distribution (related to Figure 5).
Size distribution of small RNA mapping to the Arabidopsis TAIR10 genome; the abundance of reads per million (RPM) of each size class was calculated for each source of data. The x axis indicates the small RNA size (from 18 to 34 nt) and the y axis indicates its abundance. Small RNA distribution for total RNA samples (A) and small RNA size distribution for AGO1 IP samples (B).

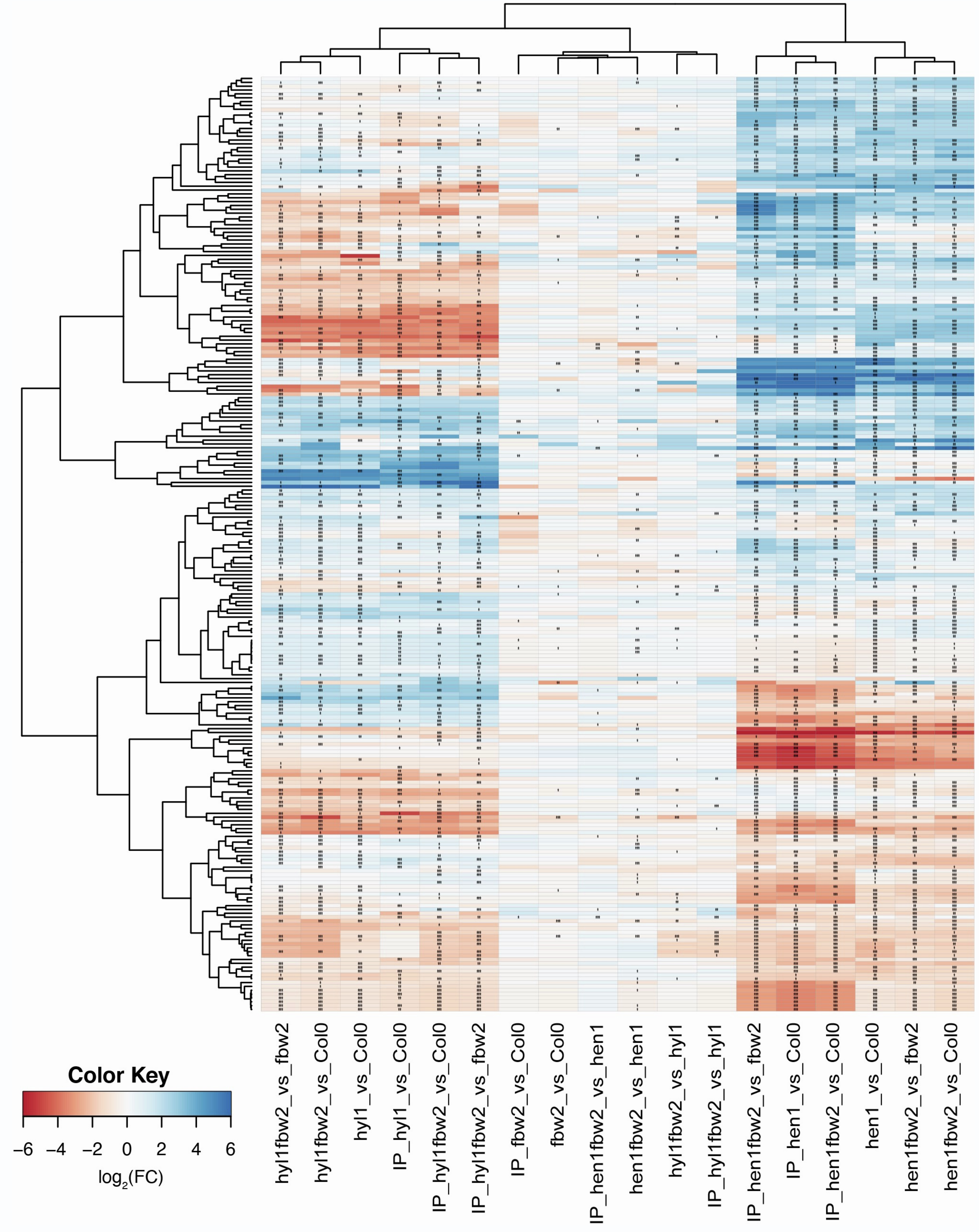

Figure S7. ***FBW2*** **mutation has a minimal effect in the miRNA population (related to Figure 5).**
Relative abundance of miRNA with significant differential expression in single and double mutants compared to wild-type (Col-0) and single mutants; the relative abundance is expressed as a heat map (see Key at the bottom), with the samples being compared indicated below each heat map (*Q value # 0.05, **Q value # 0.01 and **Q value #0.001).

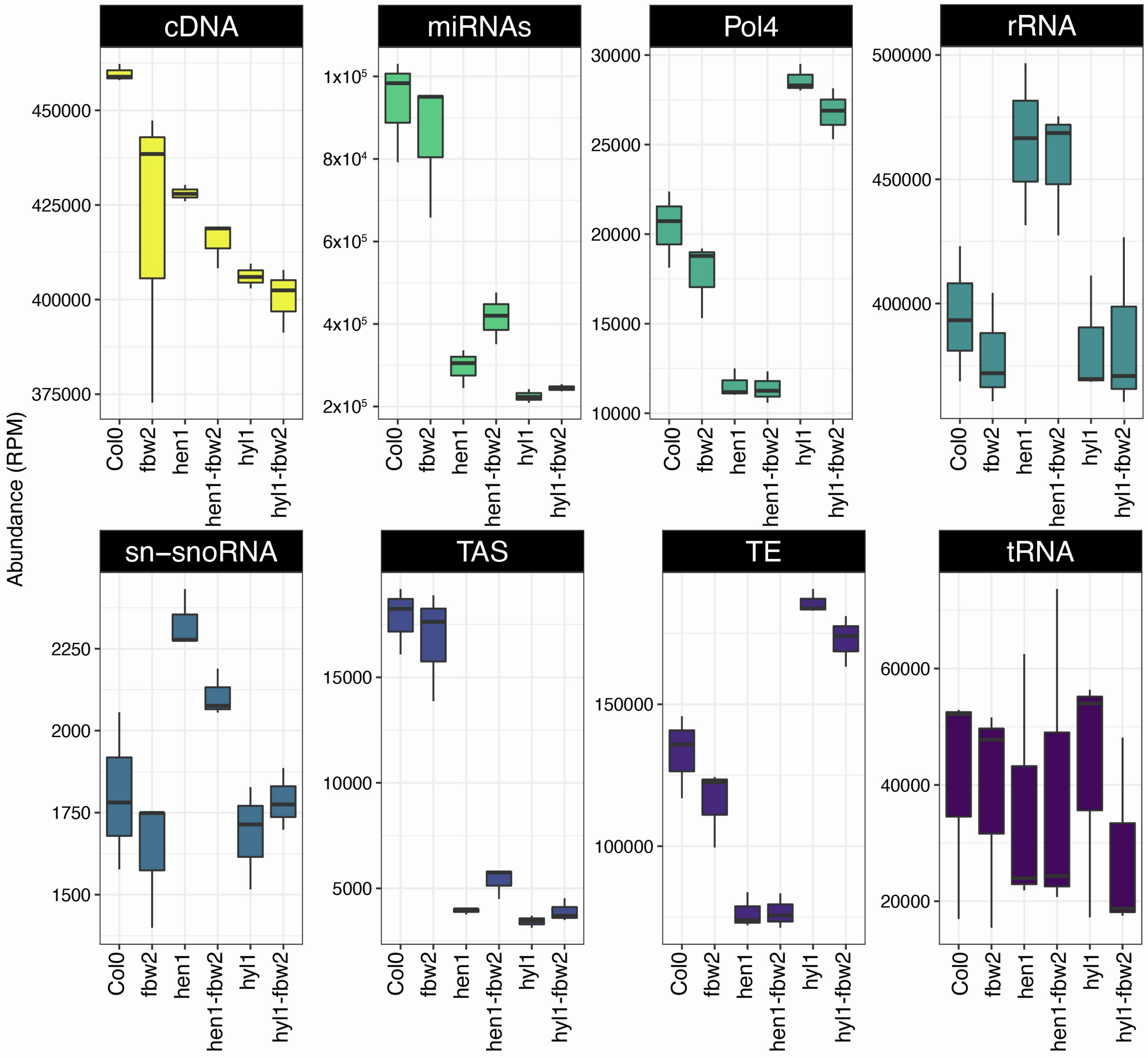

Figure S8. Pol4- and TE-derived sRNA reads are enriched in *hyl1 fbw2* double mutant, while no significant differences are observed in *fbw2* single mutants (related to Figure 5).
Boxplot representing the abundance of reads (in reads per million) mapping to eight different features of the Arabidopsis genome TAIR 10 for total RNA samples. These include the following from left to right: cDNA; mature miRNA; siRNA precursors dependent on Pol4; ribosomal RNA (rRNA); small nuclear and small nucleolar RNA (snRNA and snoRNA); TAS precursors; Transposable elements (TEs); tRNA-derived sRNA (tRNA); RPM, reads per million. Three biological replicates are included for each sample.

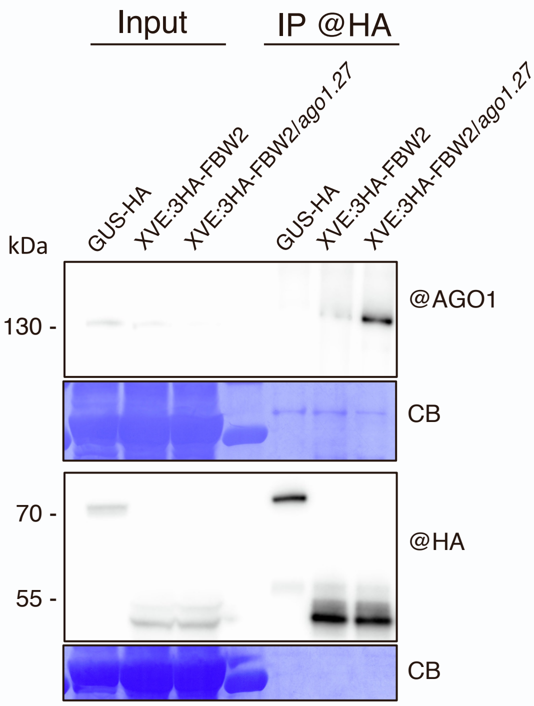

Figure S9. FBW2 interacts with AGO1 and exhibit a higher affinity toward the mutated form AGO1-27 (related to Figure 2 and 3).

Western blot of protein extracts from 7 day-old seedlings expressing XVE:3HA-FBW2 in Col-0 or *ago1-27* genetic backgrounds. 3HA-FBW2 was immunoprecipitated with anti-HA beads (µMACS HA Isolation Kit) after an induction of expression in liquid MS medium supplemented with β-Es (15µM) for 5 hours followed by 18 hours with by β-Es (15 µM) together with MLN4924 (25 µM). Protein extracts from 7 day-old seedlings expressing 35S-GUS-HA was used as a control for anti-HA immunoprecipitation. The "@" symbol indicates hybridization with the corresponding antibodies. Despite a lower accumulation level of AGO1-27 (input), the protein is more efficiently coprecipitated in FBW2 IPs than WT AGO1.
