# Supplementary material for: The Arabidopsis F-box protein FBW2 targets AGO1 for degradation to prevent spurious loading of illegitimate small RNA
Source: Cell Rep. 2022 Apr 13;39(2):110671. doi: 10.1016/j.celrep.2022.110671 (PMC9035678; doi:10.1016/j.celrep.2022.110671)
Supplement: Document S2. Article plus supplemental information [file mmc6.pdf]

Cell Reports

Article

The Arabidopsis F-box protein FBW2 targets AGO1 for degradation to prevent spurious loading of illegitimate small RNA

Graphical abstract

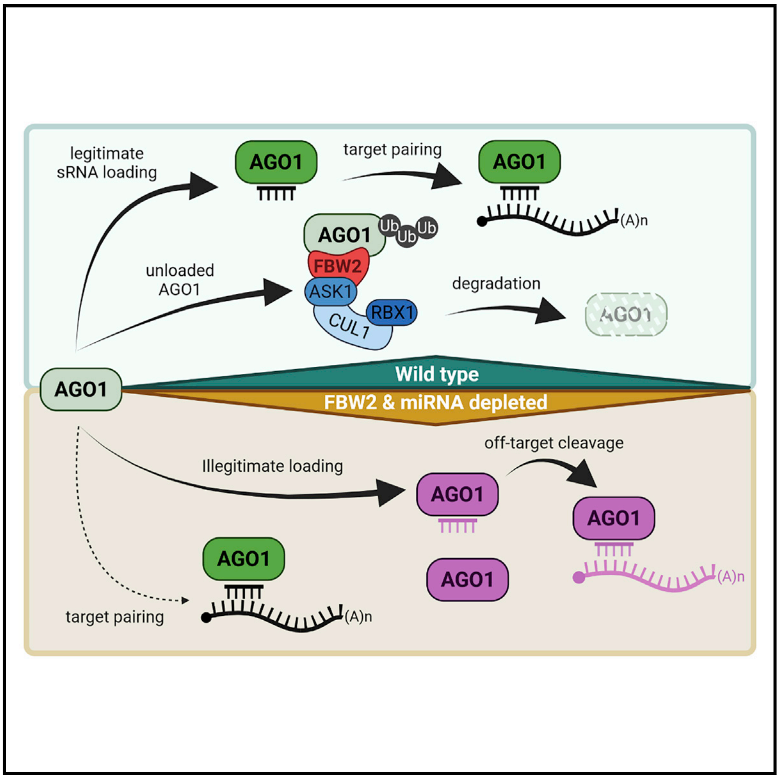

Highlights

- FBW2 assembles an SCF complex targeting specifically empty AGO1
- *FBW2* mutation leads to AGO1 accumulation and increased RNA silencing activity
- Impaired sRNA accumulation in *fbw2* results in AGO1 illegitimate loading
- FBW2-mediated AGO1 proteolysis avoids off-target cleavage

Authors

Thibaut Hacquard, Marion Clavel, Patricia Baldrich, ..., Hervé Vaucheret, Blake C. Meyers, Pascal Genschik

Correspondence

pascal.genschik@ibmp-cnrs.unistra.fr

In brief

Argonaute proteins, main effectors of RNA silencing, remain stable only when they are loaded with small RNA. Hacquard et al. show that the E3 ubiquitin ligase component FBW2 triggers empty AGO1 degradation in Arabidopsis. Interfering with this mechanism leads to AGO1 spurious loading of illegitimate small RNA and off-target cleavage.

Hacquard et al., 2022, Cell Reports *39*, 110671
April 12, 2022 © 2022 The Author(s).
https://doi.org/10.1016/j.celrep.2022.110671

Cell Reports

CellPress
OPEN ACCESS

Article

The Arabidopsis F-box protein FBW2 targets AGO1 for degradation to prevent spurious loading of illegitimate small RNA

Thibaut Hacquard,[1,9] Marion Clavel,[1,6,9] Patricia Baldrich,[2,9] Esther Lechner,[1] Imma Pérez-Salamó,[1] Mikhail Schepetilnikov,[1] Benoît Derrien,[1,7] Marieke Dubois,[1,8] Philippe Hammann,[3] Lauriane Kuhn,[3] Danaé Brun,[1] Nathalie Bouteiller,[4] Nicolas Baumberger,[1] Hervé Vaucheret,[4] Blake C. Meyers,[2,5] and Pascal Genschik[1,*]

[1]Institut de biologie moléculaire des plantes, CNRS, Université de Strasbourg, 12, rue du Général Zimmer, 67084 Strasbourg, France

[2]Donald Danforth Plant Science Center, Saint Louis 63132, MO, USA

[3]Plateforme Protéomique Strasbourg Esplanade du CNRS, Université de Strasbourg, 67084 Strasbourg, France

[4]Institut Jean-Pierre Bourgin, INRAE, AgroParisTech, Université Paris-Saclay, 78000 Versailles, France

[5]Division of Plant Science and Technology, University of Missouri, Columbia, MO 65211, USA

[6]Present address: Gregor Mendel Institute, Austrian Academy of Sciences, Vienna, Austria

[7]Present address: DNA SCRIPT, 75014 Paris, France

[8]Present address: VIB-UGent Center for Plant Systems Biology, Ghent University, 9052 Ghent, Belgium

[9]These authors contributed equally

*Correspondence: pascal.genschik@ibmp-cnrs.unistra.fr

https://doi.org/10.1016/j.celrep.2022.110671

SUMMARY

RNA silencing is a conserved mechanism in eukaryotes involved in development and defense against viruses. In plants, ARGONAUTE1 (AGO1) protein plays a central role in both microRNA- and small interfering RNA-directed silencing, and its expression is regulated at multiple levels. Here, we report that the F-box protein FBW2 assembles an SCF complex that selectively targets for proteolysis AGO1 when it is unloaded and mutated. Although *FBW2* loss of function does not lead to strong growth or developmental defects, it significantly increases RNA-silencing activity. Interestingly, under conditions in which small-RNA accumulation is affected, the failure to degrade AGO1 in *fbw2* mutants becomes more deleterious for the plant. Accordingly, the non-degradable AGO1 protein assembles high-molecular-weight complexes and binds illegitimate small RNA, leading to off-target cleavage. Therefore, control of AGO1 homeostasis by FBW2 plays an important role in quality control of RNA silencing.

INTRODUCTION

In eukaryotes, RNA silencing is crucial for development and plays major roles in response to the environment, including pathogens, as well as in the control of transposable elements. This pathway involves processing of double-stranded (ds)RNA by the RNase III enzyme Dicer into small RNA (sRNA) of 21 to 24 nucleotides in length (Ghildiyal and Zamore, 2009). These sRNA are known to associate with Argonaute (AGO) proteins to form RNA-induced silencing complexes (RISCs) (Meister, 2013; Poulsen et al., 2013). RISCs are programmed by the bound sRNA to specifically interact with transcripts based on sequence complementarity, resulting in their down-regulation. Plant sRNA falls into two broad categories (Axtell, 2013). The first consists of microRNA (miRNA), which are excised from stem-loop structures arising from non-coding *MIR* genes and act by post-transcriptionally repressing the levels of mRNA to which they are partly complementary. The second category encompasses so-called siRNA, which are processed from long double-stranded RNA arising from a variety of sources (transposons, endogenous inverted repeats, viral RNA, transgenes) and act as repressor of expression, either transcriptionally or post-transcriptionally, by mediating RNA cleavage and/or translational repression.

The *Arabidopsis thaliana* (hereafter referred to as Arabidopsis) genome encodes 10 Argonaute paralogs (Vaucheret, 2008) that all have a similar domain organization and ability to bind to sRNA, although the nature and sequence of the sRNA bound by different AGOs vary greatly. Both genetic and biochemical analyses have revealed that AGO1 plays a central role in both miRNA- and siRNA-directed silencing (Mi et al., 2008). Hence, AGO1 loaded with miRNA mediates endonucleolytic cleavage of target transcripts (Baumberger and Baulcombe, 2005), but a fraction of transcripts can also undergo repression of protein translation (Brodersen et al., 2008; Li et al., 2013). By its ability to bind virus-derived siRNA (vsiRNA), AGO1 is also an important player in plant antiviral silencing (Azevedo et al., 2010; Morel et al., 2002).

Previous work revealed that viral suppressor of RNA silencing (VSR) proteins P0 from poleroviruses encode F-box proteins that hijack the host S phase kinase-associated protein 1 (SKP1)-cullin 1 (CUL1)-F-box protein (SCF) ubiquitin-protein ligase (E3) to promote AGO1 degradation (Baumberger et al., 2007;

This is an open access article under the CC BY license (http://creativecommons.org/licenses/by/4.0/).

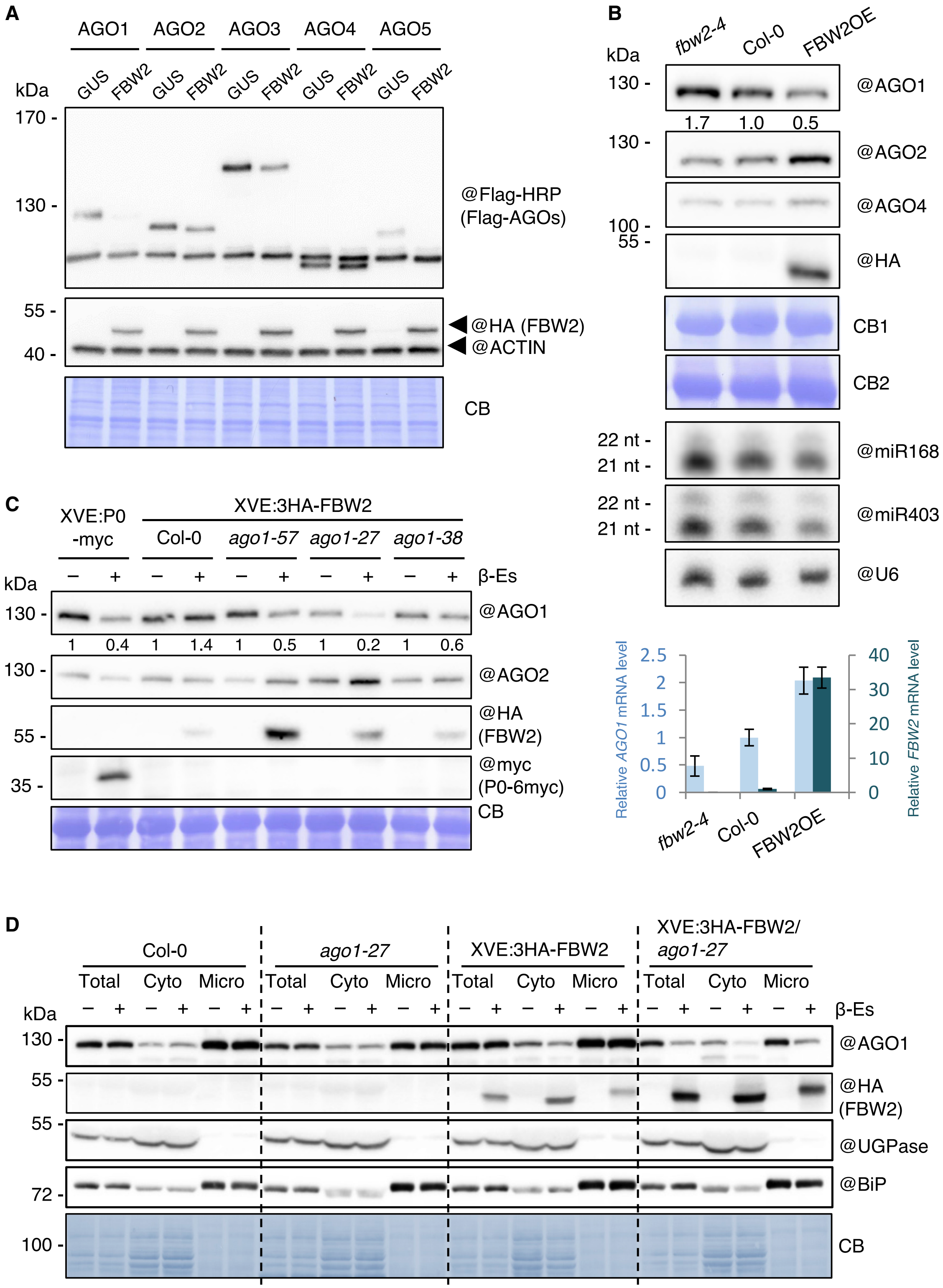

Figure 1. FBW2 triggers preferentially the degradation of AGO1

(A) FBW2 effectively induces AGO1 degradation and to lesser extent other AGOs (based on two biological replicates). Different Arabidopsis Flag-AGO proteins, under the control of the CaMV 35S promoter, were transiently expressed in *N. benthamiana* leaves in the absence (35S:GUS) or presence of FBW2 (35S:3HA-FBW2). Proteins were extracted 72 h after agro-infiltration, and AGO proteins were detected by western blot with Flag antibodies. Probing with the ACTIN antibody and Coomassie blue (CB) staining were used as loading controls.

(legend continued on next page)

Bortolamiol et al., 2007; Csorba et al., 2010; Pazhouhandeh et al., 2006). P0 triggers the vacuolar degradation of membrane-bound AGO1 via an autophagy-related process (Derrien et al., 2012; Michaeli et al., 2019), while VSRs of other viruses can also promote AGO1 degradation by a different pathway involving the proteasome (Chiu et al., 2010). Beyond manipulations of AGO1 turnover by VSRs, still little is known about post-translational regulations of AGO1 in a non-viral context. Nonetheless, in plants and metazoans, it was shown that mutations affecting miRNA biogenesis and/or accumulation and thus disturbing RISC assembly result in AGO protein turnover, suggesting that the underlying mechanisms contribute to their normal cellular homeostasis (Derrien et al., 2012; Martinez and Gregory, 2013; Smibert et al., 2013) How metazoan AGO proteins are degraded is also not well understood. For instance, it was shown that the inhibition of HSP90 function triggered the degradation of unloaded human Ago1 and Ago2 proteins (Johnston et al., 2010), an effect that could be alleviated, at least partially, by the proteasome inhibitor MG132. Human Ago2 is also subjected to degradation as a miRNA-free entity by selective autophagy (Gibbings et al., 2012). Nevertheless, for both degradation pathways of AGO proteins, the identity of the ubiquitin E3 ligase(s) involved remained unclear until recently. A hint came with the identification of a RING-type E3 ubiquitin ligase from *Drosophila* named Iruka, which preferentially binds and ubiquitylates empty Ago1 (Kobayashi et al., 2019). Moreover, in mammals, it was recently shown that extensive miRNA-target complementarity can trigger AGO proteasomal decay by another class of E3 ubiquitin ligase, exposing miRNA for degradation (Han et al., 2020; Shi et al., 2020).

In Arabidopsis, one candidate F-box protein that controls AGO1 protein homeostasis is FBW2. FBW2 interacts with several Skp1-like (ASK) proteins in yeast two-hybrid interactions and was initially identified by a genetic suppressor screen of a null allele of *SQUINT* (*SQN*), encoding a cyclophilin-40 chaperone acting as a positive regulator of AGO1 activity (Earley et al., 2010; Risseeuw et al., 2003). While *fbw2* mutant plants did not show a strong increase of endogenous AGO1 protein, likely because of the miR168-AGO1 feedback loop (Mallory and Vaucheret, 2010), overexpression of *FBW2* in transgenic Arabidopsis lines reduced AGO1 protein level (Earley et al., 2010). Loss of *FBW2* and over-expression of *FBW2* both affect AGO1 protein levels without affecting the *AGO1* transcript, suggesting that FBW2 regulates AGO1 post-transcriptionally. Moreover, under standard growth conditions, *fbw2* mutant plants exhibited no visible alteration in development, thus raising the question of the physiological role of this F-box protein (Earley et al., 2010). Interestingly, it was recently shown that *FBW2* is transcriptionally repressed by CURLY LEAF (CLF), encoding a subunit of the polycomb repressor complex 2 (PRC2) and that this regulation may be important for AGO1 protein homeostasis when plants are exposed to UV radiation (Ré et al., 2019).

In the present work, we show that, *in planta*, FBW2 does not induce the degradation of all AGOs equally, but preferentially targets AGO1. Our results indicate that FBW2 plays a critical role to maintain AGO1 proteostasis by preferentially degrading its unloaded form. Interestingly, in mutant plants lacking *FBW2* and which are impaired in sRNA accumulation, stabilized AGO1 further worsens their phenotype. Hence, we show that the non-degradable AGO1 protein assembles high molecular complexes and binds illegitimate sRNA, leading to the cleavage of different target genes. Our studies identify a mechanism to avoid AGO1 spurious loading of sRNA, which could conditionally become detrimental for cells.

RESULTS

FBW2 targets for degradation both soluble and membrane-bound AGO1

To better understand how FBW2 is involved in the control of AGO protein homeostasis, we first tested its ability to degrade different AGO proteins in a transient-expression assay. Arabidopsis AGO1, AGO2, AGO3, AGO4, and AGO5, representative members of the three AGO phylogenetic clades (Vaucheret, 2008), were tagged with a Flag tag and transiently expressed in the presence of 3HA-FBW2 (3× human influenza hemagglutinin (HA)-epitope at the N terminus of FBW2) or GUS (as a control) in *Nicotiana benthamiana* (*N. benthamiana*) leaves, and the level of each AGO protein was assessed with the Flag antibody (Figure 1A). In this assay, 3HA-FBW2 was able to degrade AGO1 and AGO5 and to a lesser extent AGO2 and AGO3, but not AGO4, which was insensitive to the F-box protein. Note that AGO5 belongs to the same phylogenetic clade as AGO1, suggesting that members of this clade are better substrates for FBW2.

(B) FBW2 overexpression leads to partial degradation of AGO1 (based on two biological replicates). Top: Immunoblot analysis of AGO1, AGO2, and AGO4 protein contents in the *fbw2-4* mutant allele, in Col-0 and in FBW2OE line (35S:3HA-FBW2 line 10). Seedlings grown on MS medium were harvested at 8 days, and protein extracts were analyzed by immunoblotting of AGOs using specific antibodies and of FBW2 using the HA antibody. CB staining was used as loading controls (CB1 corresponds to AGO1 and CB2 to 3HA-FBW2). The AGO1 signal was quantified by ImageJ, normalized to the corresponding CB. Numbers are indicated below the panel as relative to Col-0 set at 1.0. Middle: sRNA gel blot analysis of the steady-state accumulation of the indicated miRNAs taken from the same material as above. U6 RNA level was used as a loading control. Bottom: RT-qPCR analysis of *AGO1* and *FBW2* transcript levels in the *fbw2-4*, Col-0, and FBW2OE line. Total RNA samples were extracted from the same material as above. Bars indicate the mean expression of three technical replicates, and error bars indicate the SD.
(C) Western blot of protein extracts from 7-day-old seedlings XVE-P0-myc and XVE:3HA-FBW2 crossed with the specified *ago1* mutants and grown on MS medium supplemented with DMSO (−) or β-Es (10 μM; +) (based on two biological replicates). CB staining was used as loading control, and @ indicates hybridization with the corresponding antibodies.
(D) FBW2 promotes the degradation of both soluble and membrane-bound compromised pool of AGO1 in the *ago1-27* background (based on one biological replicate). Immunoblot analysis of the FBW2-mediated AGO1 subcellular degradation in the total protein extract (Total), soluble (Cyto), and membranous (Micro) fractions prepared from 7-day-old XVE:3HA-FBW2 β-Es-inducible lines in the background of Col-0 and *ago-1-27* grown *in vitro* on 1/2MS agar plates supplemented with 0.1% DMSO (−) or 10 μM β-Es (+). Cellular fractions were probed with specific antibodies against Arabidopsis AGO1, cytoplasmic UGPase enzyme, ER luminal binding protein BiP, and HA tag. CB staining served as a loading control. Note that 3HA-FBW2 expression level is higher in the *ago1-27* mutant background.

Next, we aimed to constitutively express tagged 3HA-FBW2 in Arabidopsis. For this purpose, we first verified in *N. benthamiana* leaves that the tagged protein was as efficient as the native F-box to degrade AGO1 (Figure S1A). Among the 35S:3HA-FBW2 transgenic lines selected (hereafter named FBW2OE lines) we identified several of them showing variable levels of 3HA-FBW2 protein expression (Figure S1B) and we continued with one of them (line #10), exhibiting the highest expression level. In this line, we observed a significant decrease in AGO1 protein despite a higher level of its transcript (Figure 1B). A phenotypic examination of the line showed a reduction of leaf growth and an increase in the number of lateral roots, whereas the *fbw2-4* knockout line did not produce a visible phenotype under standard growth conditions (Figures S1C and S1D). Notably, none of our 35S:3HA-FBW2 transgenic lines produced a phenotype similar to *ago1* hypomorphic mutant alleles as previously reported (Earley et al., 2010). This was also the case for transgenic 35S:FBW2 Arabidopsis lines expressing untagged FBW2 (not shown). Therefore, FBW2 only poorly promotes AGO1 degradation *in planta*, where the main AGO1 pool is assumed loaded with sRNA.

In parallel, we also generated Arabidopsis transgenic lines in which 3HA-FBW2 was expressed under the control of the β-estradiol (β-Es)-inducible promoter XVE (Zuo et al., 2000), in wild-type (WT) Col-0 and in different *ago1* mutant backgrounds, including *ago1-38* mutated in the N-terminal part of AGO1 leading to reduced membrane association (Brodersen et al., 2012), *ago1*-57 mutated in the AGO1 DUF1785 affecting sRNA duplex unwinding (Derrien et al., 2018) and *ago1-27* mutated in the AGO1 PIWI domain and impairing translational repression (Brodersen et al., 2008; Li et al., 2016) (Figure 1C). Of particular interest was the *ago1-57* mutation, which abrogates SCF-dependent P0 interaction with AGO1 (Derrien et al., 2018), as we wondered whether this mutation would also affect FBW2-mediated AGO1 degradation. However, we observed that AGO1-57 protein was degraded by FBW2, suggesting that the two F-box proteins do not share the same degron in AGO1. It is noteworthy that, among these different AGO1 mutations, the AGO1-27 protein was the most susceptible to FBW2-mediated degradation, which cannot be solely explained by increased FBW2 expression (Figure 1C).

To address the question of the specificity of FBW2 toward other Arabidopsis AGO proteins, we monitored the protein levels of AGO2 and AGO4 in our FBW2 overexpressor lines. In contrast to the effect of *FBW2* on the steady-state level of AGO1, both AGO2 and AGO4 were insensitive to the degradation activity of the F-box protein (Figures 1B and 1C). Instead, AGO2 protein levels were even increased when FBW2 was overexpressed, and this might be attributed to the partial degradation of AGO1, which in association with miR403 targets AGO2 transcript (Allen et al., 2005). Accordingly, the levels of both miR168 and miR403 were decreased in FBW2OE seedlings, and *AGO1* transcript level was upregulated (Figure 1B, middle and bottom). Conversely, in the *fbw2-4*-null mutant, AGO1 protein steady-state level was slightly increased, indicating that FBW2 contributes to maintain AGO1 protein homeostasis under normal growing conditions.

Finally, we wondered whether FBW2 acts on a specific cellular pool of AGO1. Indeed, previous research has shown that AGO1 appears in membrane-free (soluble) and membrane-bound (especially associated with the ER) forms (Brodersen et al., 2012; Li et al., 2013; Michaeli et al., 2019). We therefore evaluated AGO1 protein level in soluble and microsomal fractions upon β-Es-inducible expression of FBW2 in WT and *ago1-27* backgrounds. As expected, the ER marker (BiP) was enriched in the microsomal fraction, whereas the cytosolic enzyme UDP-glucose pyrophosphorylase (UGPase) was absent yet enriched in the soluble fraction (Figure 1D). Whereas in WT the FBW2-mediated AGO1 degradation was mainly visible in the soluble fraction, the abundance of the mutated AGO1-27 protein decreased in both soluble and microsomal fractions after β-Es treatment. From these results, we conclude that FBW2 associates with both soluble and membrane-bound AGO1, to trigger its degradation.

FBW2 assembles an SCF complex that interacts with AGO1

Next, we investigated whether FBW2 is able to interact with AGO1 *in planta*. We first examined the subcellular localization of both proteins. The coding sequence of FBW2 was fused to the Venus fluorescent protein at its N terminus and put under the control of its own promoter (pFBW2:Venus-FBW2) and co-expressed with cyan fluorescent protein (CFP)-AGO1 in *N. benthamiana* leaves. Confocal imaging revealed the co-localization of both proteins in the cytosol (Figure 2A). Note that the Venus-FBW2 protein was functional, as it caused the degradation of CFP-AGO1 in this assay. Next, we immunoprecipitated (IP) 3HA-FBW2 from Arabidopsis plants and could show that the F-box was able to efficiently pull down endogenous AGO1 in the presence of MLN4924 (Figure 2B), a drug that inhibits CUL1 neddylation (Hakenjos et al., 2011). Moreover, all components of the SCF (CUL1, ASK1 and RBX1) were also pulled down in the IP, indicating that FBW2 forms an SCF-type ubiquitin E3 ligase complex *in planta*. To further identify the interaction network of FBW2, we immunoprecipitated the F-box protein when expressed in Col-0 and in the *ago1-27* mutant, as this background showed an efficient degradation rate of AGO1, and performed mass spectrometry analysis. Proteins significantly enriched in the FBW2 IP were highlighted by a statistical analysis, calculating normalized fold changes and adjusted p values (Figure 2C and Table S1). As expected, proteins of the SCF complex are predominantly enriched, such as CUL1, the CUL-like protein 1, and RUB1, as well as ASK1, ASK20, ASK21, and the FBW2 target AGO1. AGO5 and AGO10, belonging to the same phylogenetic clade as AGO1, were also found enriched in the IP. Moreover, a significant group of proteins co-purifying with FBW2 consists of molecular chaperones, including heat shock proteins (mainly Hsp70, Hsp80, and Hsp90) and DNAJ homologs J2/J3, for which functions of some of them have previously been linked to AGO1 (Earley and Poethig, 2011; Iki et al., 2010, 2012; Sjögren et al., 2018). We also identified most proteasomal subunits, pointing to an important function of this pathway for FBW2 and/or AGO1 proteolysis.

Modulating FBW2 level impacts transgene S-PTGS efficiency

As FBW2 destabilizes AGO1, we investigated its activity in suppressing RNA silencing. At first, the effect of FBW2 on inverted-repeat post-transcriptional gene silencing (IR-PTGS)

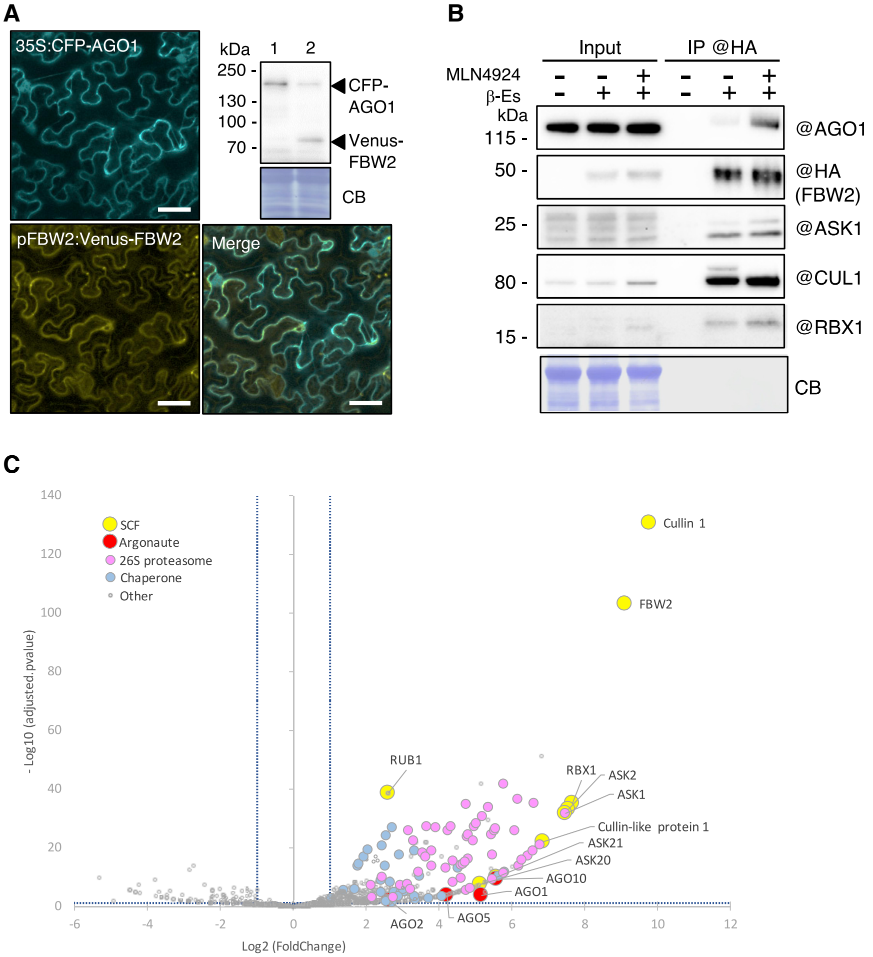

Figure 2. FBW2 assembles an SCF complex and interacts with AGO1 *in planta*

(A) Subcellular localization of CFP-AGO1 and Venus-FBW2 by confocal microscopy. Co-infiltration of 4-week-old *N. benthamiana* leaves with agrobacteria-harboring binary vectors for the expression of fluorescent-tagged protein constructs. Bacteria were infiltrated at an OD of 0.1. Pictures were taken and sampled 3 days later. For confocal microscopy imaging, CFP and Venus were excited at 458 and 514 nm, respectively. Emission signals were recovered between 465 and 510 nm for the CFP and 520 and 596 nm for the Venus. Scale bars, 40 μm. Immunodetection using GFP antibodies of protein extracts from agro-infiltrated leaves with 35S:CFP-AGO1 and pFBW2:Venus-FBW2 (lane 2) constructs is included. Expression of GUS (lane 1) served as negative control. CB staining was used as a loading control.

(B) FBW2 assembles an SCF complex and interacts *in planta* with AGO1 (based on two biological replicates; see also Figure S9). Western blot of protein extracts from 10-day-old XVE:3HA-FBW2 seedlings. 3HA-FBW2 was immunoprecipitated with anti-HA antibodies after an overnight induction of expression in liquid MS medium supplemented with DMSO (−) or β-Es (10 μM, +). 3HA-FBW2 co-immunoprecipitates with SCF components, ASK1, CUL1, and RBX1. Blocking the SCF activity with the drug MLN4924 further allowed co-immunoprecipitation of AGO1. @ indicates hybridization with the corresponding antibodies.

(C) FBW2 interactome revealed by immunoprecipitation and mass spectrometry. We compared eight samples (4 samples of FBW2OE and 4 samples of FBW2OE/ago1-27) from two independent biological replicates to seven control samples. Volcano plot shows the enrichment of proteins co-purified with HA-tagged FBW2 bait compared with Col-0 controls. The y and x axes display log values from adjusted p values and fold changes, respectively. The horizontal dashed line indicates the threshold above which proteins are significantly enriched (adjusted p values < 0.05). The vertical dashed lines indicate the fold change thresholds for FBW2-enriched proteins ($log_2 > 1$) or Col-0-enriched proteins ($log_2 < -1$). Four color-coded functional clusters are highlighted in the case of proteins enriched in the FBW2 coIP samples. The source data are available in Table S1.

was tested. For this, we used a patch assay in which a *GFP* transgene is transiently expressed in *N. benthamiana* and its silencing is triggered by a *GFFG* inverted-repeat RNA (Himber et al., 2003). In this assay, *Nicotiana tabacum* AGO1 N-terminally fused to a Flag tag was co-expressed to monitor its protein level. As expected, when the patches were infiltrated with two strong VSRs, P0 and P19 (Csorba et al., 2015), a bright-green GFP fluorescence signal was detected (Figure 3A, left). In contrast to these VSRs, FBW2 had only a weak impact on IR-PTGS triggered by the *GFFG* RNA. Quantification of the GFP fluorescence showed ±10% increased fluorescence of the silenced *GFP* when FBW2 was co-expressed (Figure 3A, right), indicating that FBW2 acts as a weak suppressor of IR-PTGS in this system. Accordingly, tobacco AGO1 is only partially destabilized by FBW2 (Figure 3B), suggesting that, together with other AGOs, the amount of active AGO1 in these plants is sufficient to execute IR-PTGS.

Next, the effect of FBW2 on sense (S)-PTGS was tested because S-PTGS is more sensitive to small perturbation in AGO1 activity than IR-PTGS. Indeed, the hypomorphic *ago1-27* mutation totally impaired S-PTGS, whereas it only decreased IR-PTGS (Parent et al., 2015). Therefore, we crossed the Arabidopsis *fbw2-1* mutant with the p35S:GUS line Hc1 (Elmayan et al., 1998). This line triggers S-PTGS in only 20% of the population at each generation, thus representing a valuable sensor to precisely monitor changes in silencing efficiency. While as expected 20% of silencing was reached in the Col-0 background, we observed that 66% of the *fbw2-1* plants were silenced (n = 96; Figure 3C). This increase in S-PTGS is consistent with the slight increase in AGO1 protein level observed in *fbw2* loss-of-function mutants (Figure 1B). It is also consistent with the fact that a higher increase in AGO1 protein level is necessary to elevate Hc1 S-PTGS frequency up to 100%, for example in 4mAGO1 plants, which express a miR168-resistant form of the AGO1 mRNA (Figure 3C; [Martínez De Alba et al., 2011]). We also generated three independent Hc1 lines overexpressing FBW2. To avoid potential interference between p35S:GUS and p35S:FBW2 transgenes, we used RFP-tagged FBW2 expressed

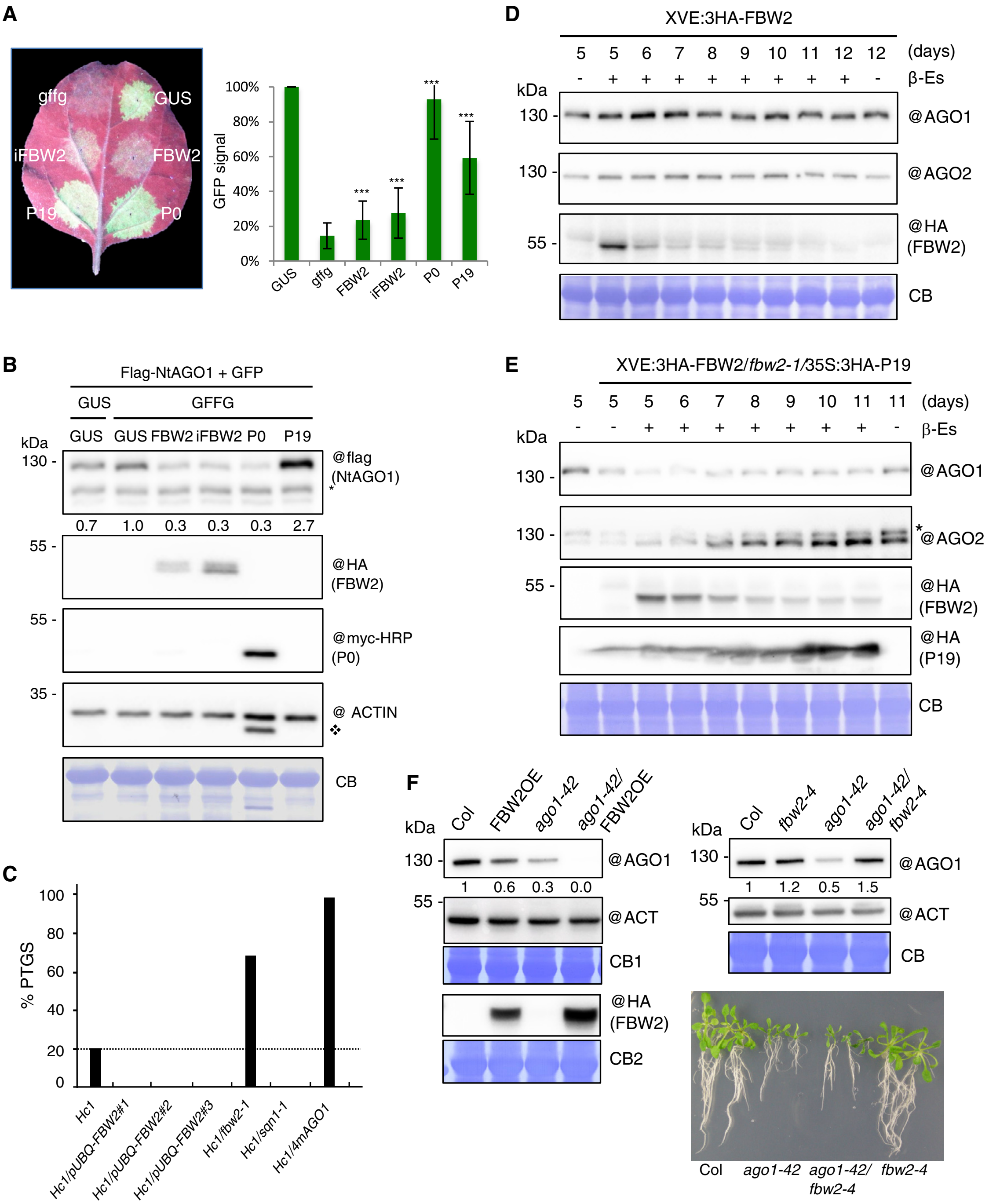

Figure 3. Function of FBW2 in RNA silencing

(A) FBW2 is a weak endogenous suppressor of RNA silencing (based on two biological replicates). Left: picture of a *N. benthamiana* leaf 72 h after infiltration with agrobacteria harboring a 35S:Flag-NtAGO1 and a 35S:GFP construct plus either the following constructs: 35S:GUS or 35S:GFFG (GFP mRNA hairpin) together with either 35S:3HA-FBW2 (only the coding sequence), 35S:3HA-iFBW2 (coding sequence including an intron), 35S:P0-6myc, or 35S:P19. Right: the intensity of GFP signal in the infiltration area was measured with an Ettan DIGE imager (GE healthcare) and normalized to the GFP control condition. ***$p < 0.001$ (t test) compared with *GFFG*.

(B) Western blot of protein extracts from tissues sampled 72 h after agro-infiltration (shown in [A]). CB staining and ACTIN protein level were used as a loading control. @ indicates hybridization with the corresponding antibodies. NtAGO1 signal was quantified by ImageJ, normalized to the corresponding CB. Numbers are indicated below the panel as relative to the control set at 1.0. *Non-specific band; ❖remaining signal from P0-6myc hybridization.

(legend continued on next page)

under the control of the pUBQ10 promoter (Grefen et al., 2010). Analysis of the HC1/pUBQ-FBW2 lines showed a clear inhibition of S-PTGS Figure 3C), which is an opposite phenotype to the *fbw2-1* mutation, and which mimics the effect of the *sqn-1* mutation (Figure 3C). This result, therefore, confirms the observation that FBW2 overexpression decreased S-PTGS of the p35S:GUS line L1 (Earley et al., 2010).

FBW2 targets preferentially AGO1 when its loading is compromised

We noted that the degradation of AGO1 by FBW2 was more effective in transient-expression assays than in stable transformed lines (Figures 1A–1D and 3D). A possible explanation of this phenomenon could be that most AGO1 is still unloaded when transiently expressed (Csorba et al., 2010), suggesting a preference of FBW2 for this form. To further address this question, we transiently co-expressed *AGO1* and *FBW2* with or without a construct carrying the inverse repeat of *GFFG*, which is known to produce functional siRNA (Himber et al., 2003). We reasoned that transient co-expression of the *GFFG* construct with AGO1 would foster its loading and could thus protect it from FBW2. However, AGO1 degradation by FBW2 was found only slightly attenuated in presence of *GFFG* (Figures S2A and S2B). By contrast, when we co-expressed the VSR P19 that specifically binds 19-21-nucleotide double-stranded sRNA (Csorba et al., 2015) and could increase the unloaded pool of AGO1, the degradation of AGO1 was more efficient. To further support this observation, we also constitutively overexpressed the P19 protein in the Arabidopsis XVE:3HA-FBW2/*fbw2-1* mutant background to deplete at least a fraction of the pool of endogenous sRNA. In agreement with the transient expression assays, AGO1 degradation by FBW2 became more effective when P19 was co-expressed than in the Col-0 background (Figures 3D and 3E). Note also the enhanced AGO2 protein level upon FBW2-mediated AGO1 depletion, likely resulting from the suppression of the negative regulation on *AGO2* transcript by AGO1 associated with miR403 (Allen et al., 2005).

To further provide evidence that the unloaded form of AGO1 is efficiently degraded by FBW2, we took advantage of the Arabidopsis *ago1-42* mutant allele, which exhibits a PAZ domain point mutation preventing sRNA loading (Devers et al., 2020). The AGO1-42 mutant protein is strongly impaired in loading 21-/22-nucleotide siRNA and miRNA sequences, and, notably, its protein level was shown markedly reduced if compared with WT, possibly because of an increased degradation rate (Devers et al., 2020). Interestingly, FBW2 overexpression entirely degraded AGO1-42 protein, and conversely, when we introduced the *fbw2* mutation in this genetic background, we fully restored the AGO1-42 protein level, supporting that the SCF^{FBW2} is the main E3 ubiquitin ligase targeting unloaded AGO1 (Figure 3F). In agreement with a defective loading of sRNA, the restabilized AGO1-42 protein in *fbw2-4* was unable to rescue the mutant phenotype.

Finally, we also examined Arabidopsis mutants affecting the production or stability of sRNA to further investigate the possible impact of AGO1 loading on its degradability by FBW2. Therefore, we crossed *fbw2-4* and *FBW2OE* mutant lines with *hyl1-2* and *hen1-6* mutants. DRB1/HYL1 mediates the processing of most miRNA precursors (Kurihara et al., 2006), whereas the RNA methyltransferase HEN1 is critical for sRNA stability (Li et al., 2005; Ren et al., 2014). To investigate the impact of *fbw2-4* and *FBW2OE* in the different genotypes on plant growth, we measured the rosette size of plants grown both on soil and *in vitro* (Figures 4A and S3A). Overexpression of *FBW2* in both mutant backgrounds revealed stronger growth defects than in the single mutants (Figures 4A and S3A). At the molecular level, these phenotypes correlated well with decreased AGO1 protein levels (Figure 4B), indicating that under conditions in which sRNA accumulation is affected AGO1 becomes more prone to degradation by FBW2.

Stabilized AGO1 in mutants impaired in sRNA accumulation is deleterious for plant development

In line with the previous report of Earley et al. (2010), we observed that AGO1 protein level is at least partially restored in *hyl1-2 fbw2-4* and *hen1-6 fbw2-4* double mutants (Figures 4B and S3B). Strikingly, despite this increased amount of AGO1 protein, we noticed that the growth and developmental phenotype of these double-mutant plants was significantly exacerbated compared with the single mutants, suggesting that the stabilized AGO1 protein became somehow toxic (Figures 4A and S3). We noticed that the growth retardation observed *in vitro* of the double mutants *hyl1-2 fbw2-4* and *hen1-6 fbw2-4* versus single mutants was significantly enhanced when plants were grown on soil and also with time. Rosette areas of both double mutants were smaller, and leaves exhibited a strong up-curling (Figure S3C). The worsening of the phenotype of the double mutants was also visible at the reproductive stage, with a clear decrease in fertility. Indeed, *hyl1-2 fbw2-4* and *hen1-6 fbw2-4* seed production was significantly reduced compared with the single *hyl1-2*

(C) Modulating FBW2 level impacts transgene S-PTGS efficiency. GUS activity was measured in leaves of 8-week-old plants of the indicated genotypes. For each genotype, 96 plants (from three biological replicates of 32 plants each) were analyzed. S-PTGS efficiency is expressed as the percentage of plants exhibiting GUS activity below 50 FLUO/min/μg.

(D and E) Kinetic analysis of transgenic Arabidopsis Col-0 XVE:3HA-FBW2 (D) (based on three biological replicates) and XVE:3HA-FBW2/*fbw2*-1/35S:3HA-P19 (E) lines (based on one biological replicate). Western blots of protein extracts from 5- to 11- or 12-day-old seedlings grown on MS medium supplemented with DMSO (−) or β-Es (10 μM,+). Note that since P19 is a powerful silencing suppressor; it allowed maintaining the expression of 3HA-FBW2 for a longer time. CB staining was used as loading control, and the "@" symbol indicates hybridization with the corresponding antibodies.

(F) FBW2-mediated degradation of the AGO1-42 mutant protein impaired in sRNA loading (based on two biological replicates). Immunoblot analysis of AGO1 protein contents in the *ago1-42* mutant background when *FBW2* is overexpressed (left) or mutated (right). Seedlings grown on MS medium were harvested at 15 days, and protein extracts were analyzed by immunoblotting using AGO1- and ACTIN-specific antibodies and the HA antibody for detecting FBW2. CB staining was used as loading controls (CB1 corresponds to AGO1 and ACTIN and CB2 to 3HA-FBW2 at the left). AGO1 signal was quantified by ImageJ and normalized to the corresponding ACTIN signal. Numbers below panels are indicated as relative to Col-0 set at 1.0. Bottom right: pictures of 25-day-old *in vitro*-grown seedlings of the indicated genotypes.

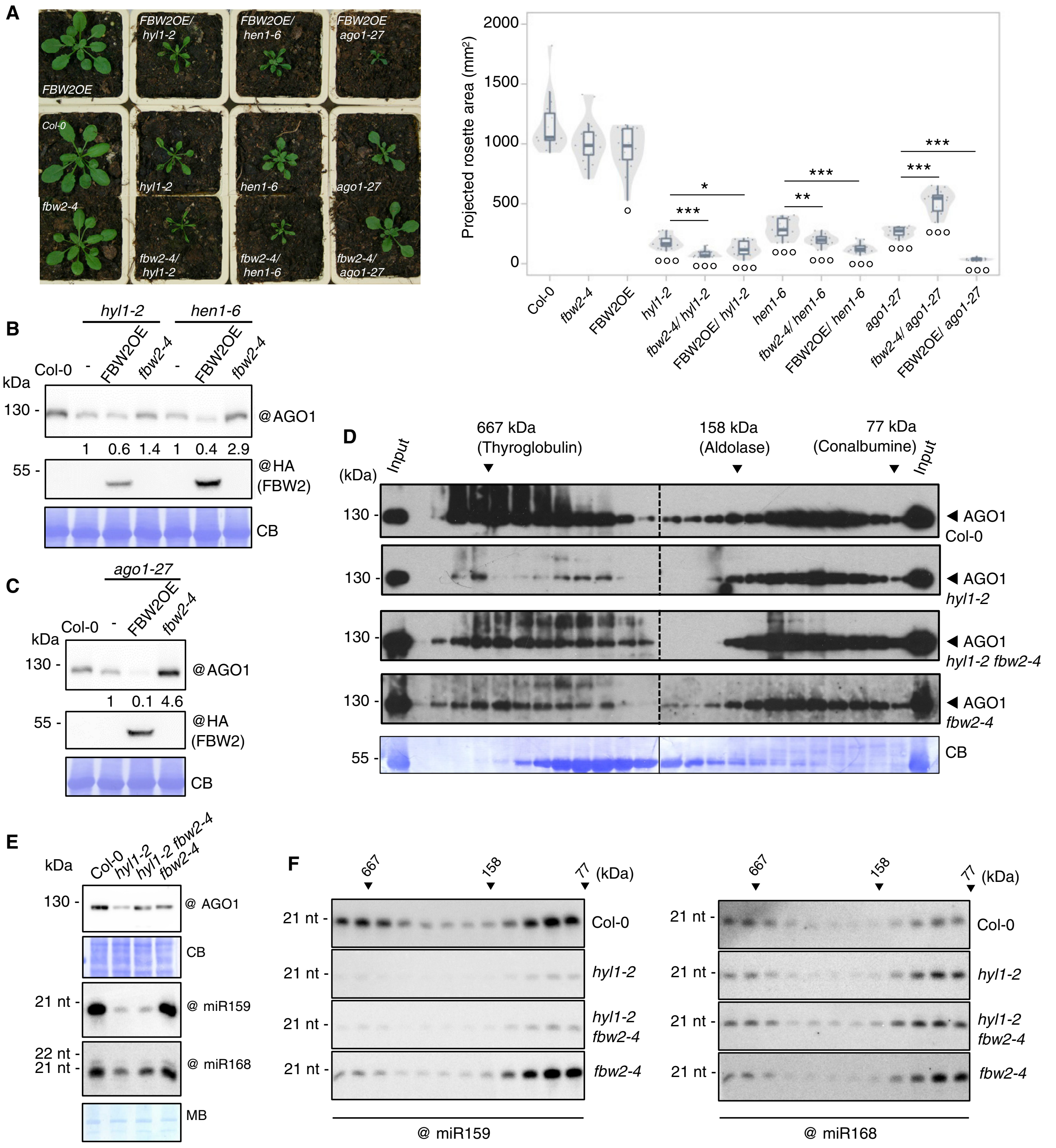

Figure 4. Effects of *FBW2* overexpression or loss of function in silencing mutants restores high-molecular-weight AGO1 complexes

(A) Representative pictures of 27-day-old plants of Col-0, *hyl1-2*, *hen1-6*, *ago1-27*, and their crosses with *fbw2-4* or 35S:3HA-FBW2 (FBW2OE) as indicated (based on two biological replicates; see also Figure S3A). Right: quantitative analysis of the experiment represented on the left, with n > 12 plants per genotype. °p < 0.05 and °°°p < 0.001 (Student's t test) compared with Col-0. *p < 0.05, **p < 0.01, and ***p < 0.001 (Student's t test) compared with the corresponding single mutant.

(B and C) Western blot of protein extracts from the same seedlings as indicated in Figure S3A (for biological replicates see Figures 5 and S3–S5). CB staining was used as loading control. AGO1 signal was quantified by ImageJ, normalized to the corresponding CB. Numbers below the panel indicate relative to the corresponding mutants (*hyl1-2*, *hen1-6* and *ago1-27*, respectively) set at 1.0. @ indicates hybridization with the corresponding antibodies.

(legend continued on next page)

and *hen1-6* mutants (Figure S3D). This strongly contrasts with the situation of *ago1-27 fbw2-4* double mutant for which the increased AGO1-27 steady-state protein level (Figure 4C) at least partially rescued the growth and fertility defects of the *ago1-27* mutant (Figures 4A, S3A, S3C, and S3D).

To better understand the reason for the apparent toxicity of stabilized AGO1 in *hyl1-2* and *hen1-6* mutant backgrounds, we first investigated the behavior of the protein in the formation of protein complexes. For these experiments we chose to work with the *hyl1-2* mutant, as *hen1-6* was nearly sterile. It has been shown that AGO1-RISC complexes are present in high- and low-molecular-weight complexes (Baumberger and Baulcombe, 2005; Csorba et al., 2010), but only the low-molecular-weight complex exhibits the slicing activity, as in animals (Nykänen et al., 2001). We thus examined the molecular weight of AGO1-based RISCs in *hyl1-2 fbw2-4* seedlings by gel filtration (GF), and the elution fractions were analyzed by western blot (Figures 4D, 4E, and S4). As expected, Col-0 exhibited both high- and low-molecular-weight AGO1-based RISCs. The *fbw2-4* single mutant behaved similarly to WT Col-0, showing both types of complexes. In contrast, the *hyl1-2* single mutant mainly presented low-molecular-weight RISCs, suggesting that the high-molecular-weight AGO1 complexes depend on miRNA accumulation. Interestingly, in the *hyl1-2 fbw2-4* double mutant, at least a fraction of the high-molecular-weight AGO1 complexes were re-established. Accordingly, miR159 co-fractionated with both the low and high molecular weight AGO1-containing complexes in Col-0 and *fbw2-4* (with, however, a lower level in the high-molecular-weight fractions), whereas in *hyl1-2* and *hyl1-2 fbw2-4* miR159 was barely detected in any fraction, probably because of its impaired synthesis (Figure 4F). This observation does not hold true for a microRNA like miR168, whose abundance is only marginally affected by the loss of *HYL1* (Szarzynska et al., 2009). On the basis of these observations, we hypothesized that, when miRNA availability is compromised and AGO1 degradation is impaired, as in the *hyl1-2 fbw2-4* double mutant, unconventional AGO1-bound RNA may be incorporated in RISCs, as supported by our gel filtration assay, and may ultimately become problematic for the plant, as indicated by the more severe phenotype in *hyl1-2 fbw2-4* and *hen1-6 fbw2-4* double mutants (Figures 4A and S3).

To better characterize the global RNA-binding activity of AGO1 in *hyl1-2* versus *hyl1-2 fbw2-4*, we immunoprecipitated AGO1 from the different genetic backgrounds and indiscriminately labeled the incorporated RNA by replacing their 5′ phosphate with a radioactive one, using polynucleotide kinase (PNK) (Figures 5A and 5B). As expected, the *hyl1-2* mutant showed a reduced amount of miRNA loaded in AGO1, while the pattern of RNA associated with AGO1 in *fbw2-4* was similar to Col-0. Remarkably, the amount of 21/22-nt-long sRNA bound to AGO1 was re-established in the *hyl1-2 fbw2-4* double mutant and, in addition, AGO1 became more loaded with 24-nt-long sRNA species.

Identification of sRNA loaded into stabilized AGO1 and their targets

To get more insights into the identity of sRNA in the context of the *fbw2-4* mutation, we performed deep-sequencing analyses on total sRNA and AGO1-associated sRNA in Col-0 and five different mutant backgrounds (e.g., *fbw2-4, hyl1-2* and *hen1-6* single, and *hyl1-2 fbw2-4* and *hen1-6 fbw2-4* double mutants) (Table S2). AGO1 protein levels in the different mutants were verified beforehand by western blot (Figures S5).

As previously described, we observed that the size distribution of total sRNA is significantly altered in *hen1* and *hyl1* mutant backgrounds (Kurihara et al., 2006; Li et al., 2005; Yu, 2005; Zhai et al., 2013) (Figures S6A). However, we noted that the mutation of *fbw2-4* did not alter the size distribution in any of the three studied backgrounds, Col-0, *hen1-6*, or *hyl1-2*. The two predominant peaks at 21 and 24 nt observed in the WT and *fbw2-4* mutants completely disappeared in the *hen1-6* and *hen1-6 fbw2-4* mutants, whereas the 21-nt peak disappeared and the 24-nt peak increased in the *hyl1-2* and *hyl1-2 fbw2-4* mutants. Regarding the AGO1-associated sRNA, we observed a similar situation, with a significant decrease of 21-mers in *hen1-6* and *hen1-6 fbw2-4* mutants and a significant increase in 24-mers in *hyl1-2* and *hyl1-2 fbw2-4* mutants (Figure S6B), as observed for AGO1-associated sRNA labeling (Figure 5B). Next, we analyzed the miRNA differential accumulation in the single and double mutants compared with WT, and in the double mutants compared with single mutants (Figures 5C and S7). Very few miRNAs were differentially accumulated when we compared the *fbw2-4* mutants with the WT: only 18 miRNAs in total RNA samples and 11 in the AGO1 IP samples (Figure 5C). We also found few differentially accumulated miRNAs when we compared the double-mutant *hyl1-2 fbw2-4* with *hyl1-2* and *hen1-6 fbw2-4* with *hen1-6*, in both total RNAs and AGO1 IP samples (Figures 5C and S7).

Next, we evaluated the genomic origin of all sRNA reads in each mutant, and we focused on the category of 24-mers bound to AGO1. We mapped all the reads from each mutant to different features in the genome. As expected, we observed that the *hyl1-2 fbw2-4* and *hyl1-2* mutants accumulated lower levels of reads derived from miRNA and *trans*-acting siRNA (TAS) genes (Figure S8). However, these mutants also accumulated a higher number of sRNAs originating from Pol IV products and transposable elements (TEs), and, when we focused on the 24-nt-long sRNA, these were loaded into AGO1 (Figures 5D and S6B). Furthermore, these mutants had more rRNA-derived 24-nt sRNA loaded in AGO1 compared with

(D and E) Gel filtration analysis of AGO1-based RISC complexes in Col-0, *hyl1-2*, *hy1-2 fbw2-4*, and *fbw2-4* 13-day-old seedlings (a biological replicate is shown in Figure S4). Proteins of known molecular weight are shown on top of the blot. CB staining was used as loading control and "@" indicates hybridization with the AGO1 antibody. (E) Shown are protein and sRNA analysis of the input fraction prior to gel filtration. Methylene blue (MB) staining of the membrane was used as loading control.

(F) sRNA analysis from even fractions, spanning the same range (from the GF of [D]). For this analysis, 10μg of the RNA per lane was loaded. The "@" symbol indicates hybridization with the indicated oligonucleotide probes.

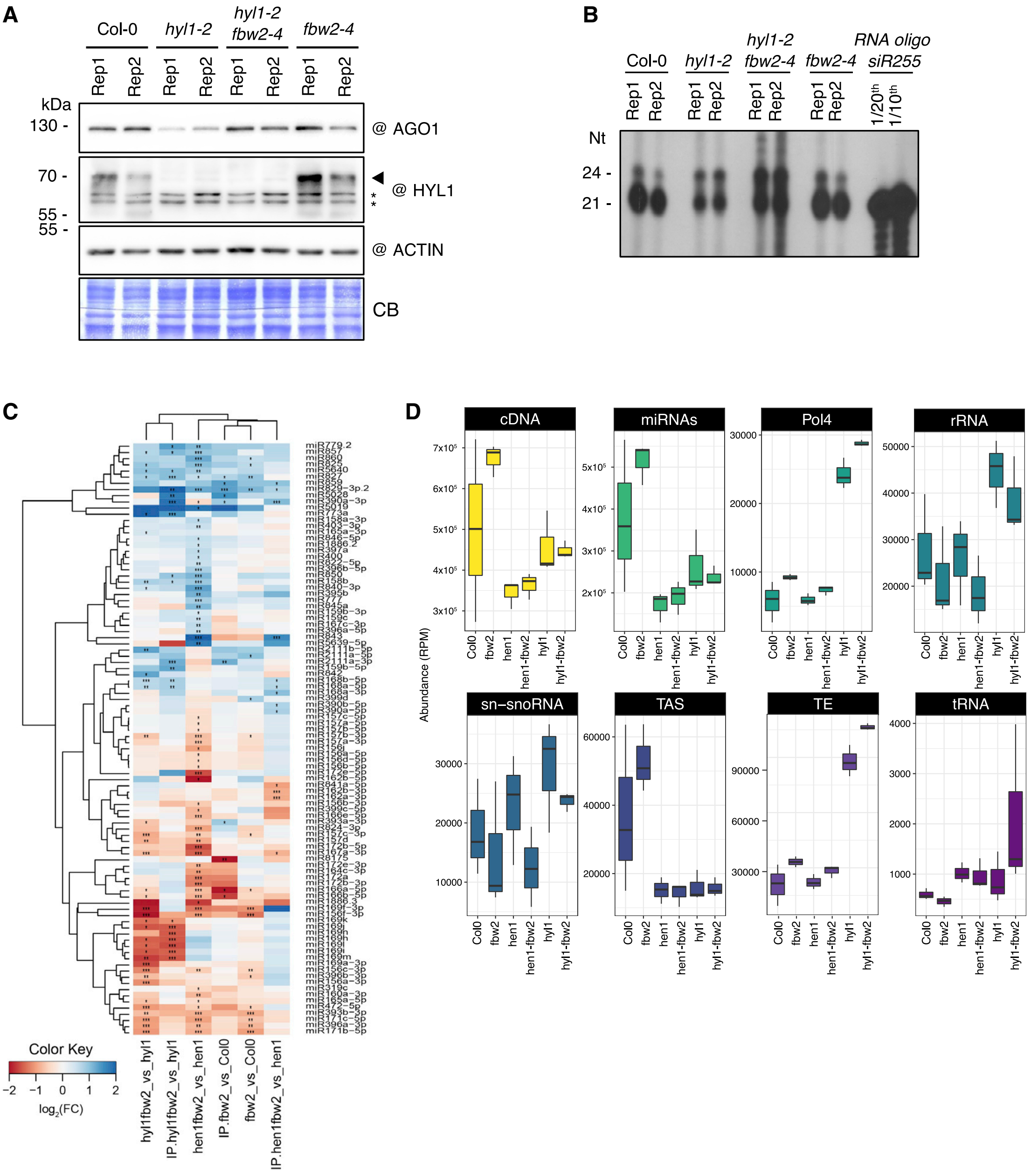

Figure 5. Loss of *FBW2* modifies AGO1 loading in *hyl1-2*

(A) Western blot of total protein extracts from 2-week-old seedlings from Col-0, *hyl1-2*, *hy1-2 fbw2-4*, and *fbw2-4* mutants. Two biological replicates (1 and 2) are shown. CB staining and ACTIN were used as loading controls, and @ indicates hybridization with the corresponding antibodies. The arrow indicates the HYL1 protein band, and * indicates aspecific cross-reacting bands.

(B) Denaturing polyacrylamide gel of sRNA from immunoprecipitated AGO1 (based on two biological replicates). RNAs from the same protein extracts shown in (A) were indiscriminately labeled by replacing their 5′ phosphate with a radioactive one using polynucleotide kinase (PNK). An oligo corresponding to the siR255 serves as control for sRNA size.

(C and D) Deep-sequencing analyses of total and AGO1-IP sRNA (performed on three biological replicates). (C) Relative abundance of miRNA in AGO1 IP samples with significant differential expression in single and double mutants compared with WT (Col-0) and single mutants; the relative abundance is expressed as a heat-map (see Key at the bottom), with the samples being compared indicated below each heatmap (*Q value #0.05, **Q value #0.01, and **Q value #0.001). (D) Boxplot representing the abundance of reads (in reads per million, RPM) mapping to eight different features of the Arabidopsis genome TAIR 10 in AGO1 IP samples. These include the following, from left to right: cDNA; mature miRNA; siRNA precursors dependent on Pol4; ribosomal RNAs (rRNAs); small nuclear and small nucleolar RNA (snRNA and snoRNA); TAS precursors; transposable elements (TEs); and tRNA-derived sRNA (tRNA).

CellPress
OPEN ACCESS

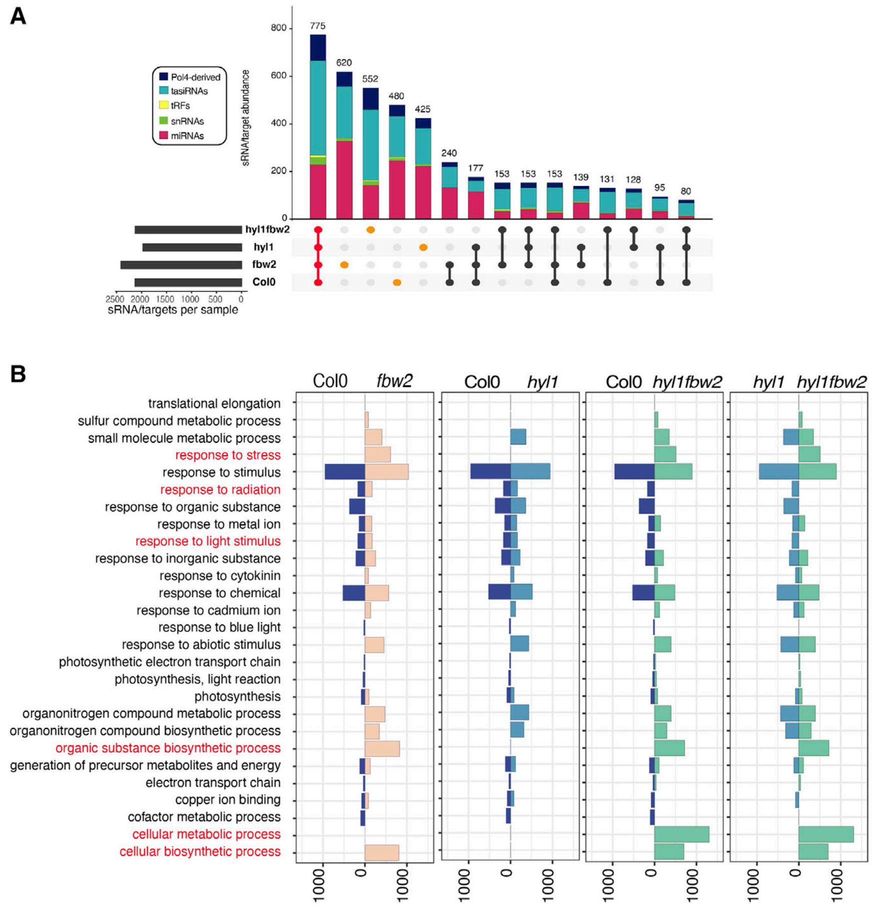

Figure 6. Loss of *FBW2* modifies sRNA-mediated targeting in *hyl1-2*

(A) Deep-sequencing analyses of PARE (performed on the same biological replicates as in Figures 5C and 5D). Visualization of the intersection of sRNA/target signatures among the different mutants, represented with an UpSet plot. Top: a vertical bar plot with the number of signatures included in each intersection, color coded into five sRNA categories, including, from top to bottom, siRNA precursors dependent on Pol4; siRNA from TAS precursors (tasiRNA); tRNA-derived sRNA (tRF); small nuclear and small nucleolar RNA (snRNA); and miRNA. Bottom left:a horizontal bar chart with the number of signatures included in each set. Bottom right indicates which intersections and their aggregates are being considered in each case. Labeled in red are the signatures common to all samples, and in orange are the signatures unique to each mutant.

(B) Vertical bar plot representing the number of genes (horizontal scale) included in each gene ontology (GO) term (vertical scale). Only significantly enriched GO terms are represented for each of the tested genotypes. From left to right are shown Col-0 versus *fbw2* mutants, Col-0 versus *hyl1* mutants, Col-0 versus *hyl1 fw2* double mutants, and *hyl1* mutants versus *hyl1 fbw2* double mutants.

WT and the other mutants (Figure 5D). We then generated parallel analysis of RNA end (PARE) libraries from the same material as for the sRNA-seq libraries (Table S2), allowing us to identify 4,301 sRNA/targets. We only considered sRNA/target signatures present in all the biological replicates, and produced by sRNA identified in AGO1 IP libraries between 19 and 24 nt in length. We observed that most of the sRNA/target signatures were shared between all the samples and were a product of miRNAs and tasiRNA (Figure 6A). In our conditions, tRNA fragments (tRF), which can act like miRNA to regulate cellular functions (Shigematsu and Kirino, 2015), and small nuclear RNA (snRNA) and small nucleolar RNA (snoRNA) did not seem to play a major role in producing sRNA with a target. We also observed a subset of specific signatures for each genotype (Figure 6A and Table S3), corresponding mostly to targets that were not present in the other genotypes. To understand how the presence or absence of these distinct or genotype-specific targets could impact the plant, we analyzed the gene ontology (GO) term enrichment in each of the tested genotypes. In our analysis, we considered only GO terms that were statistically significantly enriched when the mutants were compared with the WT (Figure 6B). We observed that the double mutants *hyl1-2 fbw2-4*, compared with Col-0 or compared with the single mutants *hyl1-2*, had an enrichment in targets belonging to GO categories such as response to stress, biosynthesis of organic substances, and cellular metabolic and biosynthetic processes, as well as a depletion of genes belonging to response to radiation and light stimulus. These results highlight clear differences in sRNA/target signatures in the double versus single mutants, which could, at least in part, explain their phenotype.

DISCUSSION

Mechanism of FBW2-mediated AGO1 degradation

Although the function of AGO proteins and their bound sRNA have been extensively studied in various biological processes across several organisms (Meister, 2013), their regulation at the post-translational level is less understood. Our laboratory and others have previously unraveled the mode of action of a viral encoded F-box protein P0 from poleroviruses, which promotes the degradation of AGO1 and thus presumably impairs RNA-based anti-viral immunity (Baumberger et al., 2007; Bortolamiol et al., 2007; Csorba et al., 2010). Because viruses usually hijack host cell machineries, it is conceivable that P0 could usurp the function of an endogenous F-box protein such as FBW2 during infection. However, although both F-box proteins target AGO proteins, our data also indicate some differences. In particular, P0 does not only mediate AGO1 turnover, but also triggers the degradation of at least AGO2 and AGO4 (Baumberger et al., 2007; Derrien et al., 2018; Trolet et al., 2019). This broad activity of P0 on several AGOs is likely key for its activity as a VSR, since

besides AGO1, at least AGO2, AGO5, and AGO7 possess antiviral activities against RNA viruses (Qu et al., 2008; Takeda et al., 2008). On the contrary, FBW2 acts specifically on AGO1 and possibly other members of its clade. Moreover, FBW2 is able to degrade the P0-resistant AGO1-57 mutant protein (Derrien et al., 2018), suggesting that the AGO1 degron motif recognized by FBW2 is distinct from the one recognized by P0.

The stability of AGO proteins has been extensively linked to their loading state. For instance, it has been shown that the inhibition of HSP90 activity, which is required for AGO loading across eukaryotes (Iki et al., 2010; Iwasaki et al., 2010), destabilizes human Ago1 and Ago2 proteins (Johnston et al., 2010). Accordingly, mutations affecting sRNA availability also destabilize AGO proteins in Arabidopsis (AGO1), *Drosophila* (Ago1) and mammals (Ago2) (Derrien et al., 2012; Martinez and Gregory, 2013; Smibert et al., 2013). Since the *fbw2* mutation restores AGO1 protein levels in various mutants, affecting sRNA biogenesis, accumulation or loading of AGO1 (Earley et al., 2010; and our work), and, most importantly, the fact that this mutation fully restores the protein level of AGO1-42, which is impaired in loading sRNA, strongly argues that this F-box protein targets for degradation the unloaded form of AGO1. This is further supported by the observation that AGO1 degradation by FBW2 was more effective in transient-expression assays, a situation in which most AGO1 is still unloaded (Csorba et al., 2010), whereas in stable transformed Arabidopsis lines, the presumably sRNA-loaded AGO1 was more resistant to this degradation. When we constitutively co-expressed P19 in Arabidopsis, which binds to both siRNA and miRNA/miRNA* duplexes, AGO1 became more susceptible to FBW2-mediated degradation. This situation is reminiscent of *Drosophila*, where unloaded Ago1 can be rescued from degradation by synthetic miRNAs but not siRNAs (Smibert et al., 2013). Interestingly it was shown that the unloaded form of *Drosophila* Ago1 is recognized and subsequently degraded by a RING-type E3 ubiquitin ligase named Iruka (Kobayashi et al., 2019). Thus, Iruka ubiquitylates Lys-514 in the L2 linker of Ago1, which is only accessible in its empty state. However, other E3 ubiquitin ligases are able to target loaded forms of AGO proteins. Hence, it was recently found that ZSWIM8, a cullin3-RING ubiquitin ligase (CRL3) adaptor protein, ubiquitylates human Ago2 when engaged with a TDMD (target-directed microRNA degradation) target, leading potentially to proteasomal degradation of the miRNA-containing complex (Han et al., 2020; Shi et al., 2020).

In line with the above-cited works, FBW2 may potentially also recognize the loaded form of AGO1. For instance, we observed that FBW2 associates not only with soluble but also with membrane-bound AGO1; this form of AGO1 could eventually be loaded and bound to its target RNA. Moreover, it was proposed that FBW2 may target AGO1 when it is bound to an mRNA under prolonged conditions, such as in the presence of non-cleavable artificial miRNA target mimics, expected to prolong the time of AGO1-target interaction (Ré et al., 2019). Though the direct involvement of FBW2 in this mechanism still remains to be demonstrated, it will be interesting to determine whether AGO1 would undergo conformational changes on its target RNA, allowing its recognition by the F-box protein. It is also tempting to speculate that some mutations, such as in *ago1-27*, leading to an increased degradation rate of the AGO1-27 protein by FBW2, would be subjected to a similar regulation. While the *ago1-27* hypomorphic allele has normal AGO1 slicer activity, it is defective in translational repression (Brodersen et al., 2008) (Li et al., 2016), although it is currently unknown why this is the case, except that the mutation seems to affect the association of miRNA to the ER membrane. Although the AGO1-27 protein can load sRNA (Li et al., 2016) (Devers et al., 2020), the over-accumulation of sRNA duplexes in the total sRNA fraction of *ago1-27* plants suggests a loading defect (Derrien et al., 2018). Thus, the *ago1-27* mutation in the PIWI domain may lead to structural constraints, leading to a protein less fit for duplex entry and/or for co-chaperone association. This mutant form might be better recognized by FBW2, which is supported by coIP experiments showing that FBW2 interaction with AGO1-27 is stronger than with WT AGO1 (Figure S9). Elucidating the molecular and cellular determinants of the FBW2-mediated degradation pathway will represent an important goal in the future.

Why is it important to degrade AGO1?

A key question to address is the physiological importance of AGO protein turnover by ubiquitin E3 ligases. In the fission yeast *Schizosaccharomyces pombe*, it was hypothesized that the accumulation of unloaded Ago1, which is involved in transcriptional silencing, might be problematic to cells as by poisoning the activity of sRNA-programmed RNA-induced transcriptional silencing (RITS) complexes (Holoch and Moazed, 2015). As discussed above, in *Drosophila*, the ubiquitin E3 ligase Iruka eliminates the empty form of Ago1 (Kobayashi et al., 2019). In that work, the authors suggested that this mechanism would be particularly relevant for dysfunctional forms of Ago1, potentially originating from translational errors or incorrect folding and locked in an empty state.

In Arabidopsis, *FBW2* loss of function does not affect plant growth or development under standard growth conditions nor the accumulation level of specific miRNA. In *fbw2* mutant plants, the AGO1 protein level was found only slightly increased, although this already significantly affected S-PTGS activity. Thus, one evident function of FBW2 is to work conjointly with the miR168 feedback loop (Mallory and Vaucheret, 2010) to maintain AGO1 homeostasis. Note that the latter appears more decisive, as the expression of miR168-resistant *AGO1* mRNA induces severe development defects in Arabidopsis (Vaucheret et al., 2004). It would be interesting to investigate the appearance and importance of both mechanisms throughout evolution in the green lineage. Another possible function for FBW2 could be its involvement in the degradation of mutated and dysfunctional forms of AGO1, as proposed for *Drosophila* (Kobayashi et al., 2019). This is supported by the observation that the AGO1-27 mutant protein was more susceptible to FBW2-mediated degradation if compared with native AGO1.

Interestingly, AGO1 post-translational control by FBW2 revealed its importance particularly under certain conditions. Indeed, when the *fbw2* mutation was combined with *hyl1-2* or *hen1-6* mutants, respectively affecting the production and stability of sRNA (Kurihara et al., 2006; Li et al., 2005; Ren et al., 2014), the stabilized AGO1 protein enhanced the growth and developmental phenotype of the single mutants. Deep-sequencing

analyses of sRNA in the different mutant background revealed that under these conditions AGO1 associates *in vivo* with sRNA, derived from categories yielding few sRNA in a WT context. Strikingly, the loading of some illegitimate sRNA in stabilized AGO1 leads to the cleavage of target genes belonging to diverse pathways including stress responses and also cellular metabolic processes. This abnormal targeting likely contributes to the enhanced phenotype observed in the *hyl-1 fbw2* double mutant. Whether the control of AGO homeostasis by E3 ubiquitin ligases to avoid off-target cleavage also operates in other organisms, such as mammals, or is unique to plants, will need further investigation.

Limitations of the study

In this paper, we showed that FBW2, as part of an SCF complex, is involved in the selective proteolysis of AGO1. However, the direct interaction of FBW2 with AGO1 is not yet demonstrated, and which structural domain of AGO1 is recognized by FBW2 needs to be determined. Another aspect that remains unclear is by which proteolytic machinery (26S proteasome versus autophagy) FBW2 mediates AGO1 degradation and which type of ubiquitin chains are involved. Finally, the physiological context of this proteolytic mechanism remains to be elucidated, but specific stress conditions involving AGO1-RISC reprogramming may shed light on it.

STAR★METHODS

Detailed methods are provided in the online version of this paper and include the following:

- KEY RESOURCES TABLE
- RESOURCE AVAILABILITY
  - Lead contact
  - Materials availability
  - Data and code availability
- EXPERIMENTAL MODEL AND SUBJECT DETAILS
- METHOD DETAILS
  - Plasmid constructions
  - Plant transformation, growth conditions and treatments with chemicals
  - S-PTGS assay
  - Confocal microscopy analysis
  - Protein immuno-precipitation assays
  - Mass spectrometry analysis, data processing and availability
  - Protein analysis and western blotting
  - Microsomal fractionation
  - Size exclusion chromatography
  - RT-qPCR and sRNA analyses by northern blotting
  - Radiolabelling of AGO1 co-purified RNA
  - Libraries preparation and high-throughput sequencing
- QUANTIFICATION AND STATISTICAL ANALYSIS

SUPPLEMENTAL INFORMATION

Supplemental information can be found online at https://doi.org/10.1016/j.celrep.2022.110671.

ACKNOWLEDGMENTS

We thank Scott Poethig for sharing material and a fruitful discussion and Thomas Potuschak for providing the GUS-HA line. P.G. acknowledges support from the European Research Council under the European Union's Seventh Framework Programme (FP7/2007-2013)/ERC advanced grant to PG agreement n° [338904] and ANR-10-LABX-0036_NETRNA, ANR-10-IDEX-0002, and ANR-17-EURE-0023 programs from the French National Research Agency. The mass spectrometry instrumentation was funded by the University of Strasbourg, IdEx "Equipement mi-lourd" 2015. Work in the Meyers lab was supported by US National Science Foundation IOS award 1842685 and resources from the Donald Danforth Plant Science Center and the University of Missouri.

AUTHOR CONTRIBUTIONS

T.H., M.C., and P.G. designed research; T.H., M.C., PB, E.L., I.P.S., M.S., BD, M.D., P.H., L.K., D.B., N.Bouteiller, N.Baumberger, and H.V. performed research; T.H., M.C., P.B., H.V., B.M., and P.G. analyzed the data; P.G. wrote the paper, with help from T.H.

DECLARATION OF INTERESTS

The authors declare no competing interests.

Received: June 16, 2021
Revised: December 14, 2021
Accepted: March 22, 2022
Published: April 12, 2022

REFERENCES

Addo-Quaye, C., Miller, W., and Axtell, M.J. (2009). CleaveLand: a pipeline for using degradome data to find cleaved small RNA targets. Bioinformatics *25*, 130–131.

Allen, E., Xie, Z., Gustafson, A.M., and Carrington, J.C. (2005). microRNA-directed phasing during trans-acting siRNA biogenesis in plants. Cell *121*, 207–221.

Axtell, M.J. (2013). Classification and comparison of small RNAs from plants. Annu. Rev. Plant Biol. *64*, 137–159.

Azevedo, J., Garcia, D., Pontier, D., Ohnesorge, S., Yu, A., Garcia, S., Braun, L., Bergdoll, M., Hakimi, M.A., Lagrange, T., et al. (2010). Argonaute quenching and global changes in Dicer homeostasis caused by a pathogen-encoded GW repeat protein. Genes Dev. *24*, 904–915.

Baumberger, N., and Baulcombe, D.C. (2005). Arabidopsis ARGONAUTE1 is an RNA Slicer that selectively recruits microRNAs and short interfering RNAs. Proc. Natl. Acad. Sci. *102*, 11928–11933.

Baumberger, N., Tsai, C.H., Lie, M., Havecker, E., and Baulcombe, D.C. (2007). The polerovirus silencing suppressor P0 targets ARGONAUTE proteins for degradation. Curr. Biol. *17*, 1609–1614.

Bortolamiol, D., Pazhouhandeh, M., Marrocco, K., Genschik, P., and Ziegler-Graff, V. (2007). The Polerovirus F box protein P0 targets ARGONAUTE1 to suppress RNA silencing. Curr. Biol. *17*, 1615–1621.

Brodersen, P., Cutts, J.A., Howard, A.D., Lewis, B.H., Squyres, S.W., Herkenhoff, K.E., Murray, B.C., Kieffer, H.H., Jakosky, B.M., Snyder, C.W., et al. (2008). Widespread translational inhibition by plant miRNAs and siRNAs. Science *320*, 1185–1190.

Brodersen, P., Sakvarelidze-Achard, L., Schaller, H., Khafif, M., Schott, G., Bendahmane, A., and Voinnet, O. (2012). Isoprenoid biosynthesis is required for miRNA function and affects membrane association of ARGONAUTE 1 in Arabidopsis. Proc. Natl. Acad. Sci. *109*, 1778–1783.

Chiu, M.H., Chen, I.H., Baulcombe, D.C., and Tsai, C.H. (2010). The silencing suppressor P25 of Potato virus X interacts with Argonaute1 and mediates its degradation through the proteasome pathway. Mol. Plant Pathol. *11*, 641–649.

Clough, S.J., and Bent, A.F. (1998). Floral dip: a simplified method for Agrobacterium-mediated transformation of Arabidopsis thaliana. Plant J. *16*, 735–743.

Csorba, T., Lózsa, R., Hutvágner, G., and Burgyán, J. (2010). Polerovirus protein P0 prevents the assembly of small RNA-containing RISC complexes and leads to degradation of ARGONAUTE1. Plant J. *62*, 463–472.

Csorba, T., Kontra, L., and Burgyán, J. (2015). Viral silencing suppressors: tools forged to fine-tune host-pathogen coexistence. Virology *479–480*, 85–103.

Derrien, B., Baumberger, N., Schepetilnikov, M., Viotti, C., De Cillia, J., Ziegler-Graff, V., Isono, E., Schumacher, K., and Genschik, P. (2012). Degradation of the antiviral component ARGONAUTE1 by the autophagy pathway. Proc. Natl. Acad. Sci. *109*, 15942–15946.

Derrien, B., Clavel, M., Baumberger, N., Iki, T., Sarazin, A., Hacquard, T., Ponce, M.R., Ziegler-Graff, V., Vaucheret, H., Micol, J.L.J.L., et al. (2018). A suppressor screen for AGO1 degradation by the viral F-box P0 protein uncovers a role for AGO DUF1785 in sRNA duplex unwinding. Plant Cell *30*, 1353–1374.

Devers, E.A., Brosnan, C.A., Sarazin, A., Albertini, D., Amsler, A.C., Brioudes, F., Jullien, P.E., Lim, P., Schott, G., and Voinnet, O. (2020). Movement and differential consumption of short interfering RNA duplexes underlie mobile RNA interference. Nat. Plants *6*, 789–799.

Dubin, M.J., Bowler, C., and Benvenuto, G. (2008). A modified gateway cloning strategy for overexpressing tagged proteins in plants. Plant Methods *4*, 1–11.

Earley, K.W., and Poethig, R.S. (2011). Binding of the cyclophilin 40 ortholog SQUINT to Hsp90 protein is required for SQUINT function in Arabidopsis. J. Biol. Chem. *286*, 38184–38189.

Earley, K., Smith, M.R., Weber, R., Gregory, B.D., and Poethig, R.S. (2010). An endogenous F-box protein regulates ARGONAUTE1 in Arabidopsis thaliana. Silence *1*, 15.

Edgar, R., Domrachev, M., and Lash, A.E. (2002). Gene Expression Omnibus: NCBI gene expression and hybridization array data repository. Nucleic Acids Res. *30*, 207–210.

Elmayan, T., Balzergue, S., Béon, F., Bourdon, V., Daubremet, J., Guénet, Y., Mourrain, P., Palauqui, J.C., Vernhettes, S., Vialle, T., et al. (1998). Arabidopsis mutants impaired in cosuppression. Plant Cell *10*, 1747–1758.

Ghildiyal, M., and Zamore, P.D. (2009). Small silencing RNAs: an expanding universe. Nat. Rev. Genet. *10*, 94–108.

Gibbings, D., Mostowy, S., Jay, F., Schwab, Y., Cossart, P., and Voinnet, O. (2012). Selective autophagy degrades DICER and AGO2 and regulates miRNA activity. Nat. Cell Biol. *14*, 1314–1321.

Grefen, C., Donald, N., Hashimoto, K., Kudla, J., Schumacher, K., and Blatt, M.R. (2010). A ubiquitin-10 promoter-based vector set for fluorescent protein tagging facilitates temporal stability and native protein distribution in transient and stable expression studies. Plant J. *64*, 355–365.

Gy, I., Gasciolli, V., Lauressergues, D., Morel, J.-B., Gombert, J., Proux, F., Proux, C., Vaucheret, H., and Mallory, A.C. (2007). Arabidopsis FIERY1, XRN2, and XRN3 are endogenous RNA silencing suppressors. Plant Cell *19*, 3451–3461.

Hakenjos, J.P., Richter, R., Dohmann, E.M.N., Katsiarimpa, A., Isono, E., and Schwechheimer, C. (2011). MLN4924 is an efficient inhibitor of NEDD8 conjugation in plants. Plant Physiol. *156*, 527–536.

Han, J., LaVigne, C.A., Jones, B.T., Zhang, H., Gillett, F., and Mendell, J.T. (2020). A ubiquitin ligase mediates target-directed microRNA decay independently of tailing and trimming. Science *370*, eabc9546.

Himber, C., Dunoyer, P., Moissiard, G., Ritzenthaler, C., and Voinnet, O. (2003). Movement of RNA silencing. EMBO J. *22*, 4523–4533.

Holoch, D., and Moazed, D. (2015). Small-RNA loading licenses Argonaute for assembly into a transcriptional silencing complex. Nat. Struct. Mol. Biol. *22*, 328–335.

Iki, T., Yoshikawa, M., Nishikiori, M., Jaudal, M.C., Matsumoto-Yokoyama, E., Mitsuhara, I., Meshi, T., and Ishikawa, M. (2010). In vitro assembly of plant RNA-induced silencing complexes facilitated by molecular chaperone HSP90. Mol. Cell *39*, 282–291.

Iki, T., Yoshikawa, M., Meshi, T., and Ishikawa, M. (2012). Cyclophilin 40 facilitates HSP90-mediated RISC assembly in plants. EMBO J. *31*, 267–278.

Incarbone, M., Ritzenthaler, C., and Dunoyer, P. (2018). Peroxisomal targeting as a sensitive tool to detect protein-small RNA interactions through *in vivo* piggybacking. Front. Plant Sci. *9*, 135.

Iwasaki, S., Kobayashi, M., Yoda, M., Sakaguchi, Y., Katsuma, S., Suzuki, T., and Tomari, Y. (2010). Hsc70/Hsp90 chaperone machinery mediates ATP-dependent RISC loading of small RNA duplexes. Mol. Cell *39*, 292–299.

Johnston, M., Geoffroy, M.-C., Sobala, A., Hay, R., and Hutvagner, G. (2010). HSP90 protein stabilizes unloaded argonaute complexes and microscopic P-bodies in human cells. Mol. Biol. Cell *21*, 1462–1469.

Karimi, M., De Meyer, B., and Hilson, P. (2005). Modular cloning in plant cells. Trends Plant Sci. *10*, 103–105.

Kobayashi, H., Shoji, K., Kiyokawa, K., Negishi, L., and Tomari, Y. (2019). Iruka eliminates dysfunctional argonaute by selective ubiquitination of its empty state. Mol. Cell *73*, 119–129.e5.

Kurihara, Y., Takashi, Y., and Watanabe, Y. (2006). The interaction between DCL1 and HYL1 is important for efficient and precise processing of pri-miRNA in plant microRNA biogenesis. RNA *12*, 206–212.

Langmead, B., and Salzberg, S.L. (2012). Fast gapped-read alignment with Bowtie 2. Nat. Methods *9*, 357–359.

Lechner, E., Xie, D., Grava, S., Pigaglio, E., Planchais, S., Murray, J.A., Parmentier, Y., Mutterer, J., Dubreucq, B., Shen, W.H., and Genschik, P. (2002). The AtRbx1 protein is part of plant SCF complexes, and its down-regulation causes severe growth and developmental defects. J. Biol. Chem. *277*, 50069–50080.

Li, J., Yang, Z., Yu, B., Liu, J., and Chen, X. (2005). Methylation protects miRNAs and siRNAs from a 3′-end uridylation activity in Arabidopsis. Curr. Biol. *15*, 1501–1507.

Li, S., Liu, L., Zhuang, X., Yu, Y., Liu, X., Cui, X., Ji, L., Pan, Z., Cao, X., Mo, B., et al. (2013). MicroRNAs inhibit the translation of target mRNAs on the endoplasmic reticulum in arabidopsis. Cell *153*, 562–574.

Li, S.S., Le, B., Ma, X., Li, S.S., You, C., Yu, Y., Zhang, B., Liu, L., Gao, L., Shi, T., et al. (2016). Biogenesis of phased siRNAS on membrane-bound polysomes in arabidopsis. Elife *5*, e22750.

Love, M.I., Huber, W., and Anders, S. (2014). Moderated estimation of fold change and dispersion for RNA-seq data with DESeq2. Genome Biol. *15*, 550.

Mallory, A., and Vaucheret, H. (2010). Form, function, and regulation of ARGONAUTE proteins. Plant Cell *22*, 3879–3889.

Martin, M. (2011). Cutadapt removes adapter sequences from high-throughput sequencing reads. EMBnet.Journal *17*, 10.

Martinez, N.J., and Gregory, R.I. (2013). Argonaute2 expression is post-transcriptionally coupled to microRNA abundance. Rna *19*, 605–612.

Martínez De Alba, A.E., Jauvion, V., Mallory, A.C., Bouteiller, N., and Vaucheret, H. (2011). The miRNA pathway limits AGO1 availability during siRNA-mediated PTGS defense against exogenous RNA. Nucleic Acids Res. *39*, 9339–9344.

Meister, G. (2013). Argonaute proteins: functional insights and emerging roles. Nat. Rev. Genet. *14*, 447–459.

Mi, S., Cai, T., Hu, Y., Chen, Y., Hodges, E., Ni, F., Wu, L., Li, S., Zhou, H., Long, C., et al. (2008). Sorting of small RNAs into arabidopsis argonaute complexes is directed by the 5′ terminal nucleotide. Cell *133*, 116–127.

Michaeli, S., Clavel, M., Lechner, E., Viotti, C., Wu, J., Dubois, M., Hacquard, T., Derrien, B., Izquierdo, E., Lecorbeiller, M., et al. (2019). The viral F-box protein P0 induces an ER-derived autophagy degradation pathway for the clearance of membrane-bound AGO1. Proc. Natl. Acad. Sci. *116*, 22872–22883.

Morel, J., Godon, C., Mourrain, P., Béclin, C., Boutet, S., Feuerbach, F., Proux, F., and Vaucheret, H. (2002). Fertile hypomorphic ARGONAUTE (ago1) mutants impaired in post-transcriptional gene silencing and virus resistance. Plant Cell *14*, 629–639.

Nakagawa, T., Ishiguro, S., and Kimura, T. (2009). Gateway vectors for plant transformation. Plant Biotechnol. *26*, 275–284.

Nykänen, A., Haley, B., and Zamore, P.D. (2001). ATP requirements and small interfering RNA structure in the RNA interference pathway. Cell *107*, 309–321.

Parent, J.S., Jauvion, V., Bouché, N., Béclin, C., Hachet, M., Zytnicki, M., and Vaucheret, H. (2015). Post-transcriptional gene silencing triggered by sense transgenes involves uncapped antisense RNA and differs from silencing intentionally triggered by antisense transgenes. Nucleic Acids Res. *43*, 8464–8475.

Pazhouhandeh, M., Dieterle, M., Marrocco, K., Lechner, E., Berry, B., Brault, V.r.V., Hemmer, O., Kretsch, T., Richards, K.E., Genschik, P., et al. (2006). F-box-like domain in the polerovirus protein P0 is required for silencing suppressor function. Proc. Natl. Acad. Sci. U S A. *103*, 1994–1999.

Perez-Riverol, Y., Csordas, A., Bai, J., Bernal-Llinares, M., Hewapathirana, S., Kundu, D.J., Inuganti, A., Griss, J., Mayer, G., Eisenacher, M., et al. (2019). The PRIDE database and related tools and resources in 2019: improving support for quantification data. Nucleic Acids Res. *47*, D442–D450.

Popov, N., Schmitt, M., Schulzeck, S., and Matthies, H. (1975). [Reliable micromethod for determination of the protein content in tissue homogenates]. Acta Biol. Med. Ger. *34*, 1441–1446.

Poulsen, C., Vaucheret, H., and Brodersen, P. (2013). Lessons on RNA silencing mechanisms in plants from eukaryotic argonaute structures. Plant Cell *25*, 22–37.

Qu, F., Ye, X., and Morris, T.J. (2008). Arabidopsis DRB4, AGO1, AGO7, and RDR6 participate in a DCL4-initiated antiviral RNA silencing pathway negatively regulated by DCL1. Proc. Natl. Acad. Sci. *105*, 14732–14737.

Ré, D.A., Cambiagno, D.A., Arce, A.L., Tomassi, A.H., Giustozzi, M., Casati, P., Ariel, F.D., and Manavella, P.A. (2019). CURLY LEAF regulates micro RNA activity by controlling ARGONAUTE 1 degradation in plants. Mol. Plant *13*, 72–87.

Ren, G., Xie, M., Zhang, S., Vinovskis, C., Chen, X., and Yu, B. (2014). Methylation protects microRNAs from an AGO1-associated activity that uridylates 5' RNA fragments generated by AGO1 cleavage. Proc. Natl. Acad. Sci. *111*, 6365–6370.

Risseeuw, E.P., Daskalchuk, T.E., Banks, T.W., Liu, E., Cotelesage, J., Hellmann, H., Estelle, M., Somers, D.E., and Crosby, W.L. (2003). Protein interaction analysis of SCF ubiquitin E3 ligase subunits from Arabidopsis. Plant J. *34*, 753–767.

Shen, W.-H., Parmentier, Y., Hellmann, H., Lechner, E., Dong, A., Masson, J., Granier, F., Lepiniec, L., Estelle, M., and Genschik, P. (2002). Null mutation of AtCUL1 causes arrest in early embryogenesis in Arabidopsis. Mol. Biol. Cell *13*, 1928–1946.

Shi, C.Y., Kingston, E.R., Kleaveland, B., Lin, D.H., Stubna, M.W., and Bartel, D.P. (2020). The ZSWIM8 ubiquitin ligase mediates target-directed microRNA degradation. Science *370*, eabc9359.

Shigematsu, M., and Kirino, Y. (2015). tRNA-derived short non-coding RNA as interacting partners of argonaute proteins. Gene Regul. Syst. Bio. *9*, 27–33.

Sjögren, L., Floris, M., Barghetti, A., Völlmy, F., Linding, R., and Brodersen, P. (2018). Farnesylated heat shock protein 40 is a component of membrane-bound RISC in Arabidopsis. J. Biol. Chem. *293*, 16608–16622.

Smibert, P., Yang, J.S., Azzam, G., Liu, J.L., and Lai, E.C. (2013). Homeostatic control of Argonaute stability by microRNA availability. Nat. Struct. Mol. Biol. *20*, 789–795.

Smith, M.R., Willmann, M.R., Wu, G., Berardini, T.Z., Möller, B., Weijers, D., and Poethig, R.S. (2009). Cyclophilin 40 is required for microRNA activity in Arabidopsis. Proc. Natl. Acad. Sci. *106*, 5424–5429.

Szarzynska, B., Sobkowiak, L., Pant, B.D., Balazadeh, S., Scheible, W.R., Mueller-Roeber, B., Jarmolowski, A., and Szweykowska-Kulinska, Z. (2009). Gene structures and processing of Arabidopsis thaliana HYL1-dependent pri-miRNAs. Nucleic Acids Res. *37*, 3083–3093.

Takeda, A., Iwasaki, S., Watanabe, T., Utsumi, M., and Watanabe, Y. (2008). The mechanism selecting the guide strand from small RNA duplexes is different among Argonaute proteins. Plant Cell Physiol *49*, 493–500.

Tian, F., Yang, D.C., Meng, Y.Q., Jin, J., and Gao, G. (2020). PlantRegMap: charting functional regulatory maps in plants. Nucleic Acids Res. *48*, D1104–D1113.

Trolet, A., Baldrich, P., Criqui, M.-C., Dubois, M., Clavel, M., Meyers, B.C., and Genschik, P. (2019). Cell cycle-dependent regulation and function of ARGONAUTE1 in plants. Plant Cell *31*, 1734–1750.

Vaucheret, H. (2008). Plant ARGONAUTES. Trends Plant Sci. *13*, 350–358.

Vaucheret, H., Vazquez, F., Crété, P., and Bartel, D.P. (2004). The action of ARGONAUTE1 in the miRNA pathway and its regulation by the miRNA pathway are crucial for plant development. Genes Dev. *18*, 1187–1197.

Wickham, H. (2016). ggplot2 (Cham: Springer International Publishing).

Yu, B. (2005). Methylation as a crucial step in plant microRNA biogenesis. Science *307*, 932–935.

Zhai, J., Zhao, Y., Simon, S.A., Huang, S., Petsch, K., Arikit, S., Pillay, M., Ji, L., Xie, M., Cao, X., et al. (2013). Plant microRNAs display differential 39 truncation and tailing modifications that are ARGONAUTE1 dependent and conserved across species. Plant Cell *25*, 2417–2428.

Zhai, J., Arikit, S., Simon, S.A., Kingham, B.F., and Meyers, B.C. (2014). Rapid construction of parallel analysis of RNA end (PARE) libraries for Illumina sequencing. Methods *67*, 84–90.

Zuo, J., Niu, Q.W., and Chua, N.H. (2000). Technical advance: an estrogen receptor-based transactivator XVE mediates highly inducible gene expression in transgenic plants. Plant J. *24*, 265–273.

STAR★METHODS

KEY RESOURCES TABLE

REAGENT or RESOURCE	SOURCE	IDENTIFIER
Antibodies		
Rabbit polyclonal anti-AGO1	Agrisera	Cat# AS09 527, RRID:AB_2224930
Rabbit polyclonal anti-AGO2	Agrisera	Cat# AS13 2682
Rabbit polyclonal anti-AGO4	Agrisera	Cat# AS09617, RRID:AB_10507623
Rabbit polyclonal anti-HYL1	Agrisera	Cat# AS06 136, RRID:AB_2233541
Rabbit polyclonal anti-ASK1	Lechner et al. (2002)	N/A
Rabbit polyclonal anti-CUL1	Shen et al. (2002)	N/A
Rabbit polyclonal anti-ROC1	Mybiosource	Cat# MBS4751158
Rabbit polyclonal anti-ACTIN	Agrisera	Cat# AS13 2640, RRID:AB_2722610
Rabbit polyclonal anti-CDC2 PSTAIRE	Santa Cruz Biotechnology	Cat# sc-53, RRID:AB_2074908
Rabbit polyclonal anti-BIP	Agrisera	Cat# AS09 481, RRID:AB_1832007
Rabbit polyclonal anti-UGPase	Agrisera	Cat# AS05 086, RRID:AB_1031827
Rabbit polyclonal anti-RFP	Chromotek	Cat# 6g6-100, RRID:AB_2631395
Mouse monoclonal anti-HA	Covance	Cat# MMS-101P-200, RRID:AB_10064068
Mouse monoclonal anti-HA	Sigma-Aldrich	Cat# H9658, RRID:AB_260092
Anti cMyc-HRP	Miltenyi	Cat# 130-092-113, RRID:AB_871937
Anti Flag-M2-HRP	Sigma-Aldrich	Cat# A8592, RRID:AB_439702
Anti GFP-HRP	Miltenyi	Cat# 130-091-833, RRID:AB_247003
Mouse monoclonal anti-GFP (JL8)	Clontech (Takara)	Cat# 632381, RRID:AB_2313808
peroxidase-conjugated goat anti-rabbit IgG	Thermo Fisher Scientific	Cat# G-21234, RRID:AB_2536530
peroxidase-conjugated goat anti-mouse IgG	Thermo Fisher Scientific	Cat# G-21040, RRID:AB_2536527
Bacterial and virus strains		
Escherichia coli	Invitrogen	Top10
Agrobacterium tumefaciens		GV3101 Pmp90
Agrobacterium tumefaciens		C58C1
Chemicals, peptides, and recombinant proteins		
Cycloheximide (use at 100 µM)	Sigma-Aldrich	C1988-1G
β–Estradiol (use at 10 or 20 µM)	Sigma-Aldrich	E8875
E64D (use at 50 or 100 µM)	Sigma-Aldrich	E8640
MLN-4924 (use at 25 µM)	Active Biochem	MLN-492
MG132 (use 10 or 100 µM)	CAS 13340782-6	Calbiochem
Bortezomib (use at 100 µM)	Selleckchem	S1013
Agar type A	Sigma Aldrich	A4550-500G
SYBR Green Master Mix	Roche	Cat N° 04707516001
ECL prime kit	GE Healthcare	Cat N° WBLUF0100
Clarity Western ECL substrate	Biorad	1705061
deoxynucleotide triphosphate (dNTP)	Promega	U1205
MS medium	Duchefa	MO255
Protease inhibitor: complete-EDTA free	Roche	04693132001
Igepal CA-630	Sigma Aldrich	I8896
Formaldehyde	Thermo Scientific	28906
µMACS HA Isolation Kit	Miltenyi Biotech	130-091-122
µ Columns	Miltenyi Biotech	130-042-701
Criterion TGX 4-15% gradient precast gels	Biorad	5671084

(*Continued on next page*)

Continued

REAGENT or RESOURCE	SOURCE	IDENTIFIER
Nupage 4–12% gradient precast gels gels	Fisher Scientific	10472322
High capacity cDNA Reverse transcriptase	Fisher Scientific	4368813
Dnase I	Fisher Scientific	10649890
T4 Polynucleotide Kinase	Promega	M4103
T4 Polynucleotide Kinase	Thermo Fisher	EK0031
Glycogen	Thermo Fisher	R0561
Syringic acid	Sigma Aldrich	S6881
4-methylumbelliferyl-β-D-glucuronide	Duchefa	M1404
Pierce Anti-HA magnetic Beads	Thermo Fisher	88836
PureProteome Protein A Magnetic Bead System	Sigma Aldrich	LSKMAGA
**Deposited data**		
mass spectrometry proteomics data deposited to the ProteomeXchange Consortium via the PRIDE [(Perez-Riverol et al., 2019)] partner repository.	Dataset identifier PXD024840 and https://doi.org/10.6019/PXD024840	N/A
Data for sRNA seq	GEO Series accession number GSE169324	N/A
Data for PARE seq	GEO Series accession number GSE169434	N/A
**Experimental models: Organisms/strains**		
Nicothiana benthamiana		N/A
Arabidopsis thaliana ecotype Colombia		N/A
ago1-27	(Morel et al., 2002)	N/A
ago1-42	(Devers et al., 2020)	N/A
ago1-57	(Derrien et al., 2018)	N/A
fbw2-1	(Earley et al., 2010)	N/A
fbw2-4		SALK_144548
hen1-6		SALK_090960
hyl1-2		SALK_064863
sqn-1	(Smith et al., 2009)	N/A
XVE :P0-myc	(Derrien et al., 2012)	N/A
Hc1	(Elmayan et al., 1998; Martínez De Alba et al., 2011)	N/A
L1	(Elmayan et al., 1998; Martínez De Alba et al., 2011)	N/A
Hc1/*sqn-1*	(Elmayan et al., 1998; Martínez De Alba et al., 2011)	N/A
Hc1/*4m*AGO1	(Elmayan et al., 1998; Martínez De Alba et al., 2011)	N/A
35S:3HA-P19	(Incarbone et al., 2018)	N/A
**Oligonucleotides**		
Primers for qPCR, see Table S4	This paper	N/A
Primers for genotyping, see Table S4	This paper	N/A
Primers for cloning, see Table S4	This paper	N/A
Probe sequence, see Table S4	This paper	N/A
**Software and algorithms**		
ImageJ version 1.45		https://imagej.nih.gov/
Lightcycler 480 software, Release 1.5.0 SP3	Roche	Cat. No. 04994884001
Cutadapt v2.9	(Martin, 2011)	N/A
Bowtie2	(Langmead and Salzberg, 2012)	N/A
DESeq2	(Love et al., 2014)	N/A
CleaveLand v4.5	(Addo-Quaye et al., 2009)	N/A

(*Continued on next page*)

Continued		
REAGENT or RESOURCE	SOURCE	IDENTIFIER
PlantRegMap tool	(Tian et al., 2020)	N/A
ggplot2	(Wickham, 2016)	N/A
Graphical abstract picture	Created with BioRender.com	N/A

RESOURCE AVAILABILITY

Lead contact

Further information and requests for resources and reagents should be directed to and will be fulfilled by the lead contact, Pascal Genschik (pascal.genschik@ibmp-cnrs.unistra.fr).

Materials availability

Transgenic plant seeds generated in this study are available from the Lead Contact on request.

Data and code availability

- Availability of the RNA-seq and proteomics data through online repertories: The mass spectrometry proteomics data have been deposited to the ProteomeXchange Consortium via the PRIDE [(Perez-Riverol et al., 2019)] partner repository with the dataset identifier PXD024840 and https://doi.org/10.6019/PXD024840. The deep sequencing data have been deposited in NCBI's Gene Expression Omnibus (Edgar et al., 2002) and are accessible through GEO Series accession number GSE169324 for sRNA seq (https://www.ncbi.nlm.nih.gov/geo/query/acc.cgi?acc=GSE169324) and GSE169434 for PARE seq (https://www.ncbi.nlm.nih.gov/geo/query/acc.cgi?acc=GSE169434).
- This paper does not report original code.
- Any additional information required to reanalyze the data reported in this work paper is available from the Lead Contact upon request.

EXPERIMENTAL MODEL AND SUBJECT DETAILS

Arabidopsis thaliana ecotype Colombia as well as *Nicotiana benthamiana* (for transient expression) were used in this study. The following Arabidopsis mutants, *ago1-27* (Morel et al., 2002), *ago1-42* (Devers et al., 2020) *ago1-57* (Derrien et al., 2018), *fbw2-1* (Earley et al., 2010), *fbw2-4* (SALK_144548), *hen1-6* (SALK_090960), *hyl1-2* (SALK_064863) and *sqn-1* (Smith et al., 2009) were used. The XVE:P0-myc, Hc1, L1 and Hc1/sqn-1 and Hc1/4mAGO1 and 35S:3HA-P19 stable lines have been described previously (Derrien et al., 2012; Elmayan et al., 1998; Martínez De Alba et al., 2011; Incarbone et al., 2018).

METHOD DETAILS

Plasmid constructions

The 35S:P19, 35S:GUS, and 35S:P0-myc, 35S:Flag-AGO1, 35S:Flag-AGO2, 35S:Flag-AGO3, 35S:Flag-AGO4, 35S:Flag-AGO5 constructs have been described in Baumberger et al., (2007). The XVE:P0-6myc, p35S-GFP, p35S-GFFG constructs have been described in Bortolamiol et al., (2007). The 35S:CFP-AGO1 construct was described in Derrien et al., (2018).

The AGO1 WT constructs (pENTRY(Zeo)-AGO1) was generated by PCR amplification from the Arabidopsis AGO1 cDNA with the oligonucleotide primers listed in Table S4. Amplicons containing the attB sites were recombined into pDONR Zeo plasmids (Invitrogen). They were then transferred into the binary vector pK7WGF2, pB7WGC2 and pH7WGF2 (Karimi et al., 2005) by Gateway LR reaction to create the final N-terminal GFP, CFP or RFP fusion placed under the regulation of the 35S promoter.

The Flag-NtAGO1 construct (pENTRY(221)-FlagNtAGO1) was amplified using primers listed in Table S4. The Flag-NtAGO1 sequence containing the attB sites was recombined into pDONR 221 plasmids (Invitrogen) and then transferred into the binary vector pB2GW7 (Karimi et al., 2005) by Gateway LR reaction to create the final NtAGO1 N-terminal Flag fusion placed under the regulation of the 35S promoter.

The 3HA-FBW2 construct was generated by PCR amplification from the FBW2 cDNA with the oligonucleotide primers listed in Table S4. Amplicons were cloned by restriction (BamHI-NotI) into pE2N plasmid (Dubin et al., 2008). Then, 3HA-FBW2 was transferred from pE2N-3HA-FBW2 into the binary vector pB2GW7 (Karimi et al., 2005) by Gateway LR reaction to create the final N-terminal 3HA fusion placed under the regulation of the 35S promoter.

The 35S:FBW2 construct was generated by PCR amplification from the FBW2 cDNA with the oligonucleotide primers listed in Table S4 and used for gateway recombination using respectively the pDONR-Zeo plasmid (Invitrogen). Then, FBW2 was transferred from pENTRY(Zeo)-FBW2 into the binary vector pB2GW7 (Karimi et al., 2005) by Gateway LR reaction to create the final FBW2 placed under the regulation of the 35S promoter (p35S: FBW2).

For microscopy analysis, we generated the pFBW2:Venus-FBW2 construct. The *FBW2* promoter, the Venus and *FBW2* coding sequences were amplified by PCR using primers listed in Table S4 and used for gateway recombination using respectively the pDONR-P4P1R, pDONR-221 and pDONR–P2RP3 plasmids (Invitrogen). Then, the pENTRY obtained: pEN-L4-PromFBW2-R1, pEN-L1-VENUS-L2 and pEN-R2-FBW2-L3 were transferred into the binary vector pH7m34GW (Karimi et al., 2005) by Gateway LR reaction to create the final FBW2 N-terminal Venus fusion placed under the regulation of the *FBW2* promoter (pFBW2:Venus-FBW2).

The FBW2 genomic construct (pENTRY(221)-iFBW2) was amplified from genomic DNA by PCR amplification using primers listed Table S4. The *FBW2* genomic sequence containing the attB sites was recombined into pDONR 221 plasmids (Invitrogen) and then transferred into the binary vector pGWB415 (Nakagawa et al., 2009) by Gateway LR reaction to create the final iFBW2 (FBW2 coding sequence including an intron) N-terminal 3HA fusion placed under the regulation of the 35S promoter.

The pUBQ:RFP-iFBW2 (also called pUBQ:FBW2) construct was obtained by Gateway LR recombination (Invitrogen) using pENTRY(221)-iFBW2 described earlier and the binary vector pUBN-RFP (Grefen et al., 2010) by Gateway LR reaction to create the final iFBW2 N-terminal RFP fusion placed under the regulation of the UBI10 promoter.

Plant transformation, growth conditions and treatments with chemicals

For transient expression in *N. benthamiana* leaves, Agrobacterium cells (GV3101 Pmp90 or C58C1) harboring the constructs of interest were grown overnight at 28°C in 10mL LB medium supplemented with antibiotics, resuspended in 10mM MgCl2 supplemented with 200mM acetosyringone at an OD of 0.3 per construct (unless otherwise specified), and incubated for 1 h at room temperature before being pressure infiltrated into leaves of 4 week-old plants. Unless otherwise specified, all agro-infiltration assays were conducted in presence of P19. Plants were maintained in growth chambers under 16 h light and 8 h dark photoperiod with a constant temperature of 22°C. Sampling and observations were performed 72 h after agro-infiltration.

T-DNA transformation of Arabidopsis plants was performed using the floral dip method (Clough and Bent, 1998). For *in vitro* culture conditions, Arabidopsis seeds were surface-sterilized using ethanol and plated on MS agar (MES-buffered MS salts medium [Duchefa, Murashige & Skoog medium inc. vitamins/MES- MO255], 1% sucrose, and 0.8% agar, pH 5.7). The seeds were then stratified for 2 days at 4°C in the dark and then transferred in 16h-light/8h-dark (20,5/17°C, 70% humidity) growth chamber, under fluorescent light (Osram Biolux 58W/965). Unless otherwise specified, seedlings were transferred in liquid MS (MES-buffered MS salts medium [Duchefa, MO255], 1% sucrose, pH 5.7) and acclimated for 24 h prior to chemical treatments.

For P0-myc and XVE:3HA-FBW2 induction during plant growth, MS-agar plates were supplemented with 10μM β-estradiol, while for mock treatment, an equal amount of DMSO was used. Plates were then handled as indicated above, and seedlings were harvested at 7 to 8 days or as indicated after sowing for protein content analysis or 9 to 10 days after sowing for aerial and root growth measurements. For kinetic induction of XVE:3HA-FBW2, seedlings were grown as indicated above for 8 to 12 days, then transferred into liquid MS medium (Duchefa, MO255) +1% sucrose in sterile conditions. Liquid MS medium was then replaced with either MS + DMSO (mock) or MS + 10μM β-estradiol.

For IP-MS experiments, Col-0, FBW2OE and FBW2OE/*ago1.27* Arabidopsis lines were grown for 8 days on MS-agar plates then transferred into liquid MS medium +20μM MLN4924 for 20 h before harvesting.

S-PTGS assay

GUS activity was quantified using crude extracts from plant leaves and monitoring the quantity of 4-methylumbelliferone products generated from the substrate 4-methylumbelliferyl-β-D-glucuronide (Duchefa) on a fluorometer (Thermo Scientific fluoroskan ascent) (Gy et al., 2007).

Confocal microscopy analysis

Confocal microscopy was performed on a LEICA TCS SP8 laser scanning microscope (Leica Microsystem) using the objective HCX APO CS 20× magnification with a numeric aperture of 0,7 without immersion. Usual excitation/detection-range parameters for CFP and Venus were 458 nm/465–510 nm and 514 nm/600–630 nm, respectively and emissions were collected using system hybrid (Hyd) detectors.

Protein immuno-precipitation assays

For immunoprecipitation of HA-FBW2, 1g of frozen plant material (10 day-old seedlings) ground to a fine powder with a mortar and pestle, resuspended in 3 volumes of IP Extraction Buffer (25mM Tris HCl, pH 7.5, 150mM NaCl, 10% glycerol, 5mM MgCl2, 0.1% Tween 20, 15mM EGTA, 10μM MG132, and 1× cOmplete^{TM} Protease Inhibitor Cocktail [Roche]) and incubated for 30 min at 8 rpm in the cold room. Insoluble material was removed by centrifugation (twice 15 min, 16 000g, 4°C). Identical amounts of crude extracts were incubated with 25μL anti-HA magnetic beads (Pierce Anti-HA magnetic Beads) (pre-washed three times in IP Extraction buffer) for 3 h at 8 rpm at room temperature. Immune complexes were washed three times in the IP extraction buffer. Elution of the immunoprecipitated proteins was performed by adding 30μL of glycine (0,2M pH 3) to the magnetic beads and transfer to a solution containing 10μL Tris HCl 1M pH 11. Before analysis on SDS-PAGE gels, 4X Laemmli loading buffer was added to a final concentration of 1X to the samples and then denatured for 5 min at 95°C.

For immunoprecipitation of endogenous AGO1, 500mg of frozen tissues (from 7 day-old seedlings) was ground to a fine powder with a mortar and pestle, resuspended in 3 volumes of crude extract buffer (50mM Tris, pH 7.5, 150mM NaCl, 10% glycerol, 5mM MgCl2, 0.1% IGEPALl, 5mM DTT, and 1x cOmplete^{TM} Protease Inhibitor Cocktail [Roche]), and incubated for 20 min at 8 rpm in the cold room. Insoluble material was removed by centrifugation (twice 15 min, 16,000g, 4°C). Identical amounts of crude extracts were incubated with prebound @AGO1 (5μg) PureProteome Protein A magnetic beads (30μL; Millipore) for 2 h at 7 rpm in the cold room. Immune complexes were washed four times in the crude extract buffer, and purified sRNA was eluted from the beads in Tri-Reagent (Sigma-Aldrich) following the manufacturer's instructions.

Mass spectrometry analysis, data processing and availability

For each IP, 1g of seedlings was ground in liquid nitrogen for 10 min in 3 mL of ice-cold lysis buffer (50mM Tris, 50mM NaCl, 0.25% IGEPAL CA-630, 2mM MgCl2, 1mM DTT, 0.375% formaldehyde, protease inhibitors (cOmplete^{TM}–EDTA free, Roche). The cross-linked protein extract was quenched 2 min with glycine to a final concentration of 200mM. The cleared supernatants were divided in two affinity purifications, incubated with magnetic microbeads coupled to HA antibodies (Miltenyi, catalogue number 130-091-122), and complexes were eluted in 100 μL of pre-warmed elution buffer (Miltenyi). Co-IP experiments were performed in two independent biological replicates with two different transgenic lines (FBW2OE and FBW2OE/ago1.27). Each biological replicate was divided into two affinity-purification replicates. In parallel control IPs were carried out with HA antibodies in Col-0.

Eluted proteins were digested with sequencing-grade trypsin (Promega, Fitchburg, MA, USA). Each sample was further analyzed by nanoLC-MS/MS on a QExactive + mass spectrometer coupled to an EASY-nanoLC-1000 (Thermo-Fisher Scientific, USA). Proteins were precipitated overnight with 5 volumes of cold 0.1 M ammonium acetate in 100% methanol. After washing twice the protein pellets with cold 0.1 M ammonium acetate in 80% methanol, proteins were further resuspended in 50 mM ammonium bicarbonate. Proteins were reduced (5mM dithiothreitol, 10 min, 95°C) and alkylated (10mM iodoacetamide, 30 min, RT, in the dark). After a quenching step (5 mM dithiothreitol), proteins were digested overnight with 150ng of sequencing-grade porcine trypsin (Promega, Fitchburg, MA, USA). The resulting vacuum-dried peptides were resuspended in water containing 0.1% (v/v) formic acid (solvent A). The peptide mixtures (500ng) were analyzed using an Easy-nanoLC-1000 system coupled to a Q-Exactive Plus mass spectrometer (Thermo-Fisher Scientific, Bremen, Germany) operating in positive mode with a nanoelectrospray source. 5μL of each sample were loaded on a C-18 precolumn (75 μm ID × 20 mm nanoViper, 3μm Acclaim PepMap; Thermo-Fisher Scientific) at 800 bars in solvent A. After desalting and concentration, the pre-column was switched online with the analytical C18 analytical column (75 μm ID × 25 cm nanoViper, 3μm Acclaim PepMap; Thermo-Fisher Scientific) equilibrated in solvent A: solvent B (95:5; v/v). Peptides were eluted at a flow rate of 300 nL/min using a gradient from 5% B to 20% B in 120 min, from 20% B to 32% B in 15min, from 32% B to 95% B in 1min and 95% B to 95% B during 24min. The Q-Exactive Plus was operated in data-dependent acquisition mode (DDA) with Xcalibur software (Thermo-Fisher Scientific). Survey MS scans were acquired at a resolution of 70K at 200 m/z (mass range 350–1250), with a maximum injection time at 100ms and an automatic gain control (AGC) set at 3×10^6. Up to 10 of the most intense multiply charged ions (≥ 2) were selected for HCD fragmentation with a normalized collision energy set at 27, at 17.5K resolution, with a maximum injection time at 100ms and AGC set at 1×10^3. A dynamic exclusion time of 10 s was applied during the peak selection process. Raw files were finally transformed into mgf files using Proteome Discoverer software (v2.0, Thermo-Fisher Scientific).

Data were searched against the TAIRv10 fasta protein sequences from *Arabidopsis thaliana* with a decoy strategy (27.282 forward protein sequences). Peptides and proteins were identified with Mascot algorithm (version 2.6.2, Matrix Science, London, UK) and data were further imported into Proline v2.0 software (http://proline.profiproteomics.fr/). Proteins were validated on Mascot pretty rank equal to 1, and 1% FDR on both peptide spectrum matches (PSM score) and protein sets (Protein Set score). The total number of MS/MS fragmentation spectra was used to quantify each protein from at least six independent biological and affinity replicates. After a DEseq2 normalization of the data matrix, the spectral count values were submitted to a negative-binomial test using an edgeR GLM regression through R (R v3.2.5). For each identified protein, an adjusted p-value (adjp) corrected by Benjamini–Hochberg was calculated, as well as a protein fold-change (FC). The results are presented in a Volcano plot using protein log2 fold changes and their corresponding adjusted (-log10adjp) to highlight upregulated and downregulated proteins.

Protein analysis and western blotting

Proteins were extracted in pre-heated (95°C) 2X Laemmli sample buffer, quantified using amido-black staining (Popov et al., 1975) and 10 to 20μg of total proteins were separated by SDS-PAGE, either on 7–12% Tris-glycine gels or gradient NuPAGE 4–12% Bis-Tris Protein Gels (Thermo Fischer) or gradient Criterion TGX gel (4–15%) (BioRad). List of antibodies and their working dilution used in this work are reported in (Table S5). For all western blots, immuno-luminescence was detected using the ECL Prime kit (GE Healthcare) or ECL Clarity (BioRad) and imaged using Fusion FX (Vilbert).

Microsomal fractionation

The crude cell extracts were prepared from 7 day-old seedlings that were ground with mortar and pestle in an ice-cold buffer containing 50mM HEPES pH 7.6, 30mM KCl, 5mM MgCl2, 5mM EGTA pH 8.0 and 250mM sucrose supplemented with freshly added 1mM DTT and Protease Inhibitor cocktail (Roche). After centrifugation at 1,000 x g for 5 min at 4°C the resulting supernatant represents the total extract (Total). The microsomal (Micro) and cytoplasmic (Cyto) fractions were collected by centrifugation of soluble cell

extract in a TLA-110 rotor (Beckman Coulter ultracentrifuge) at 100,000 x g for 30 min at 4°C. For protein analysis, fractions were precipitated with methanol/chloroform and protein pellets were dissolved in 1x Laemmli buffer.

Size exclusion chromatography

About 800mg seedlings of the indicated genotypes were extracted in 2.5 Volumes of 50mM Tris-HCl pH7.5, 150mM NaCl, 5mM MgCl2, 10% glycerol, 0.1% IGEPAL, 5mM DTT, 10μM MG132, 1X cOmplete™ Protease Inhibitor Cocktail (Roche) and left to mix on carousel for 30 min. Extracts were centrifuged 10 min at 4400 rpm in Falcon tubes, filtered through Miracloth, and filtered through a 0,2μm Minisart RC 4 syringe filter. Crude extracts were calibrated to 1.9μg/μL using the amidoblack method, and 500μL were injected in 4 separate loops. Separation was performed sequentially on a Superdex 200 10/300 increase column on an AKTA Pure system with the following settings: 500μL/min, fraction volume of 250μL collected from 7.25mL to 13mL. For proteins, half of the fractions was precipitated in 2 volumes of absolute ethanol at 4°C for 48 h. Samples were centrifuged at maximum speed for 30 min at 4°C, pellets were resuspended in 2x Laemmli buffer and treated at 95°C for 5 min. Denatured samples were separated in 7.5% acrylamide SDS PAGE gels and treated as indicated in the immunoblot section. Input samples were collected from the crude extract and analyzed separately following the same method. For RNA, half the fraction (every two fraction) was mixed with 300μL tri-Reagent and extracted as indicated. Precipitated RNA was resuspended in a final concentration of 60% formamide, 5mM EDTA, 0.05% bromophenol blue-0.05% xylene cyanol, heated a 95°C for 5 min, and separated by electrophoresis on a 15% polyacrylamide (19:1 acrylamide:bisacrylamide), 8M Urea, 0.5× TBE gel at 15 watts. Electroblotting, crosslinking and hybridization was performed as standard. Input samples were collected from the original plant material and analyzed separately following the same method.

RT-qPCR and sRNA analyses by northern blotting

For quantitative RT-PCR (qPCR), 1μg of total RNA extracted in Tri-Reagent according to the manufacturer's instruction was treated with DNaseI (Fisher Scientific) and reverse transcribed with High-Capacity cDNA Reverse Transcription Kit (Applied Biosystem). PCR was performed using gene specific primers (see Table S4) in a total volume of 10μL SYBR Green Master mix (Roche) on a LightCycler LC480 apparatus (Roche) according to the manufacturer's instructions. The mean value of three replicates was normalized using the EXP (AT4G26410), and TIP41 (AT4G34270) genes as internal controls. All primers used in qRT-PCR are listed in Table S4.

For sRNA analysis, RNA was extracted in Tri-Reagent according to the manufacturer's protocol, and aqueous phase was left in 1 volume of isopropanol over-night at −20°C, precipitated 30 min at 16000g (4°C). Pellets were rinsed in 1mL 70% ethanol and centrifuged an additional 10 min. Dry RNA pellets were resuspended in 60% deionised formamide. RNA gel blot analyses of low molecular weight RNA were performed with 10μg of total RNA. Low molecular weight RNAs were resuspended in a final concentration of 60% formamide, 5mM EDTA, 0.05% bromophenol blue- 0.05% xylene cyanol, heated a 95°C for 5 min, and separated by electrophoresis on 15% polyacrylamide gels (19:1 acrylamide:bisacrylamide) 8 M Urea, 0.5× TBE gel. Separated RNA species were electroblotted on Hybond-NX (Amersham) membrane and fixed by carbodiimide-mediated cross-linking. DNA oligonucleotides complementary to miR403, miR168, miR159 and U6 RNA (see Table S4) were 5′ end-labelled with [γ-32P]ATP using T4 polynucleotide kinase (PNK) (Promega). Hybridization was performed overnight in PerfectHyb Plus (Sigma-Aldrich) at 42°C and membranes were washed once in 2× SSC-2% SDS and twice in 1× SSC-1% SDS before exposure.

Radiolabelling of AGO1 co-purified RNA

AGO1 immunoprecipitation were performed as indicated, from 400mg of 2 week-old seedlings of the indicated genotypes. Purified RNA was eluted from the beads in tri-Reagent, as indicated, and RNA was precipitated overnight at −20°C in 50% isopropanol and 40μg of glycogen. Stabilization of AGO1 in the *hyl1-2/fbw2-4* crude extract was verified by immunoblot before proceeding with labeling. Precipitated RNA was resuspended in 10μL of ultrapure water and 5μL was [γ-32P]ATP labeled by T4 polynucleotide kinase (Thermo Fisher Scientific) for 35 min at 37°C in buffer B. Labeled RNA was Tri-Reagent extracted as indicated, the aqueous phase filtered through a G25 MicroSpin column (GE healthcare) and the flowthrough precipitated in 75% isopropanol with 40μg of glycogen overnight at −20°C. A control reaction with 90ng of siR255 (21-nt) RNA oligo was treated in an identical fashion. Labeled RNA was resuspended in a final concentration of 60% formamide-5 mM EDTA-0.05% bromophenol blue-0.05% xylene cyanol, heated a 95°C for 5 min, and separated by electrophoresis on a 15% polyacrylamide (19:1 acrylamide:bisacrylamide), 8M urea, 0.5×TBE gel at 15 watts for 230 min. The gel was wrapped in plastic and the signal was detected using FUJI medical x-ray films.

Libraries preparation and high-throughput sequencing

Total RNA samples were extracted from 1-week-old Col-0, *hyl1-2*, *hyl1-2 fbw2-4*, *hen1-6*, *hen1-6 fbw2-4* and *fbw2-4* seedlings grown on MS-agar plates using Tri-Reagent according to the manufacturer's instruction. For AGO1-loaded sRNA samples, IPs were performed as described above from 500mg of 1-week-old Arabidopsis seedlings grown on MS-agar plates. AGO1-loaded sRNA were then extracted by adding Tri-Reagent directly on the magnetic beads and extraction of RNA was then performed according to the manufacturer's instructions.

Small RNA libraries were constructed using Real-Seq-AC kit (RealSeq®-AC, USA) according to manufacturer's instructions with 500ng as starting material. Libraries were sequenced using Illumina Next-Seq 500 technology at University of Delaware (Delaware, USA). Parallel analysis of RNA end (PARE) libraries were constructed following the previously published protocol (Zhai et al., 2014) and using 20ug of total RNA as starting material. Briefly, extracted RNA was purified using Dynabeads™ mRNA Purification Kit

(ThermoFisher, #61006) to obtain polyadenylated RNA. Subsequently, we ligated a 5′ RNA adapter to the 5′ end of cleaved, single-stranded RNAs, and did a second polyA purification step. We then generated the first strand of cDNA using Superscript III Reverse Transcriptase (ThermoFisher, #18080044), and the second strand and PCR amplification using Phusion® High-Fidelity DNA Polymerase (NEB, #M0530). The product was then purified using AMPure XP beads (Beckman Coulter, #A63880), and digested with MmeI restriction enzyme (NEB, #R0637), leaving a 20 nt signature fragment with overhang ends. We then ligated duplex adapter at the 3′ end using T4 DNA ligase (NEB, #M0202T) and PAGE purify the resulting fragment. Finally, we did a PCR amplification using indexed primers from TruSeq Small RNA Library Preparation Kits (Illumina, #RS-200-0012). The final library product was again PAGE purified, pooled and send for sequencing using a HiSeq2500 instrument at the University of Vermont (Vermont, USA).

For this study, a total of 36 sRNA libraries (with at least 10 M mapped reads were ultimately used) and 12 PARE libraries were constructed. For sRNA libraries, we trimmed adapters and low-quality reads using Cutadapt v2.9 software (Martin, 2011), retaining only reads between 18- and 34-nt in length. Reads were then mapped to the Arabidopsis genome version 10 (available at www.arabidopsis.org/download/) and its corresponding TAIR10 BLAST sets for all the features, using Bowtie2 (Langmead and Salzberg, 2012). Differential accumulation was done using DESeq2 (Love et al., 2014) and all plots were generated using ggplot2 (Wickham, 2016) packages in R environment. PARE libraries were trimmed and quality checked using the same tools as sRNA libraires, and analyzed using CleaveLand v4.5 (Addo-Quaye et al., 2009). To achieve a higher reliability, we only considered sRNA/target signatures in categories 0-1-2, that are present in all the biological replicates, and produced by sRNA identified in AGO1 IP libraries between 19 and 24 nt in length. Gene Ontology (GO) analysis was performed using PlantRegMap tool (Tian et al., 2020) and the GO term enrichment tool with all the identified genes as background and p-value Bonferroni correction.

QUANTIFICATION AND STATISTICAL ANALYSIS

Analysis of leaf area and lateral roots. For leaf area analysis, seedlings were grown in soil. The seeds were stratified in water and darkness for 48 h. Plastic pots (7x7x7cm) were filled with 62g of soil (Hawita Fruhstorfer erde) and watered with tap water to reach a relative humidity of 2.2gH20/gsoil (RWC 69%). Three seeds were sown in the middle of the pot. The pots were covered with a plastic foil to maintain the humidity level and placed in a 16h-light (21°C) and 8h-darkness (18°C) regime. At 4 days after stratification (DAS), the plastic film was removed, and at 5 DAS plants were thinned out to keep one plant per pot. The pots were watered every 2 to 3 days and maintained at a relative soil humidity of 2.2gH20/gsoil. Plant size at 22 DAS was determined by dissecting every leaf and placing it from oldest to youngest on a petri dish with 1% agar. The plates were photographed and the leaf area was measured with ImageJ v1.45 (NIH; https://rsb.info.nih.gov/ij/). Ten plants per line were used.

For lateral root measurements, seedlings were grown on vertical petri dishes with MS medium as described above. After eight days of growth, the number of lateral roots of each seedling was counted under the binocular. Only macroscopically visible lateral roots were counted (stage > VIII). At least ten seedlings per line were analyzed.

For GFP fluorescence quantification (Figure 3A), GFP fluorescence emitted from the N. benthamiana agro-infiltrated leaves was quantified with a Ettan DIGE image (GE healthcare) with the parameters set for the SYPRO Ruby 1 dye (excitation filter 480/30 and emission filter 595/25) with 0.017 s exposure time.

Cell Reports, Volume *39*

Supplemental information

The Arabidopsis F-box protein FBW2 targets AGO1 for degradation to prevent spurious loading of illegitimate small RNA

Thibaut Hacquard, Marion Clavel, Patricia Baldrich, Esther Lechner, Imma Pérez-Salamó, Mikhail Schepetilnikov, Benoît Derrien, Marieke Dubois, Philippe Hammann, Lauriane Kuhn, Danaé Brun, Nathalie Bouteiller, Nicolas Baumberger, Hervé Vaucheret, Blake C. Meyers, and Pascal Genschik

Supplemental information

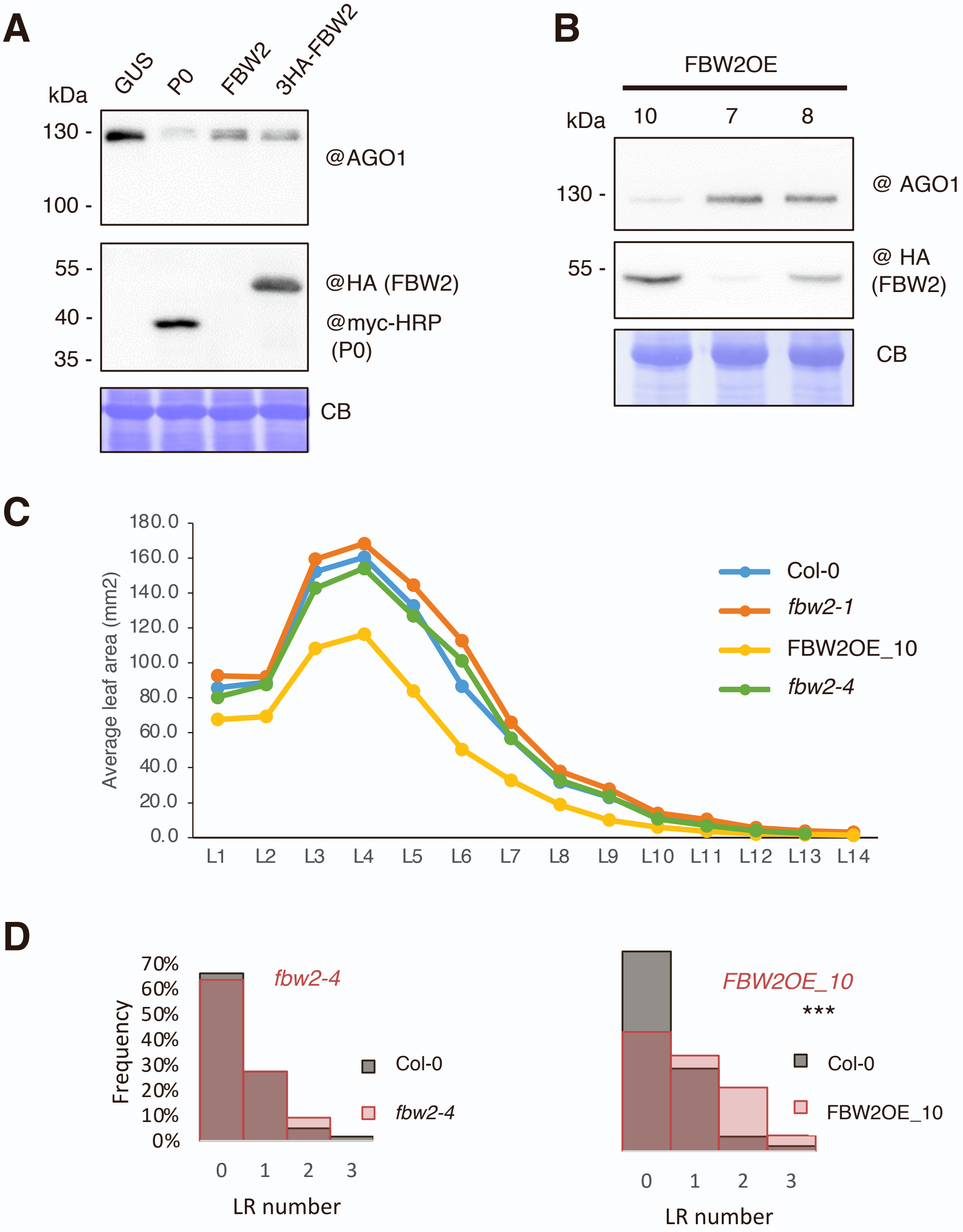

Figure S1. Overexpression of 3HA-FBW2 *in planta* and phenotypic characterization of the mutants (related to Figure 1). (**A**) Western blot of protein extracts from 4 week-old *N. benthamiana* agro-infiltrated leaves. Agrobacteria harbouring binary vectors for the expression of 35S:CFP-AGO1 combined with either 35S:GUS (control), 35S:P0-6myc, 35S:FBW2 and 35S:3HA-FBW2 constructs were infiltrated at an OD of 0,3 and tissues were sampled 3 days later. Coomassie blue (CB) staining was used as loading control. The "@" symbol indicates hybridization with the corresponding antibody.
(**B**) Three representative independent homozygous, simple insertion, FBW2OE (35S:3HA-FBW2) transgenic lines expressing variable amount of 3HA-FBW2 protein. Protein extracts from 7 day-old seedlings grown on MS medium were analysed by western blot. Coomassie blue (CB) staining was used as loading control. The "@" symbol indicates hybridization with the corresponding antibodies.
(**C**) Average area of each individual leaf, from old to young (L1-L14), of *fbw2-1*, *fbw2-4* and the FBW2OE line 10. Measurements were performed at 22 days after stratification of plants grown in soil. Ten plants were measured for each line.
(**D**) Number of lateral roots of young seedlings of *fbw2-4* and FBW2OE line 10. Lateral roots were counted at 8 days after stratification after growth *in vitro* on vertical petri dishes. Lateral roots were counted under a binocular (lateral root stage >VIII) for at least 10 plants per line. *** = P<0.001, t-test.

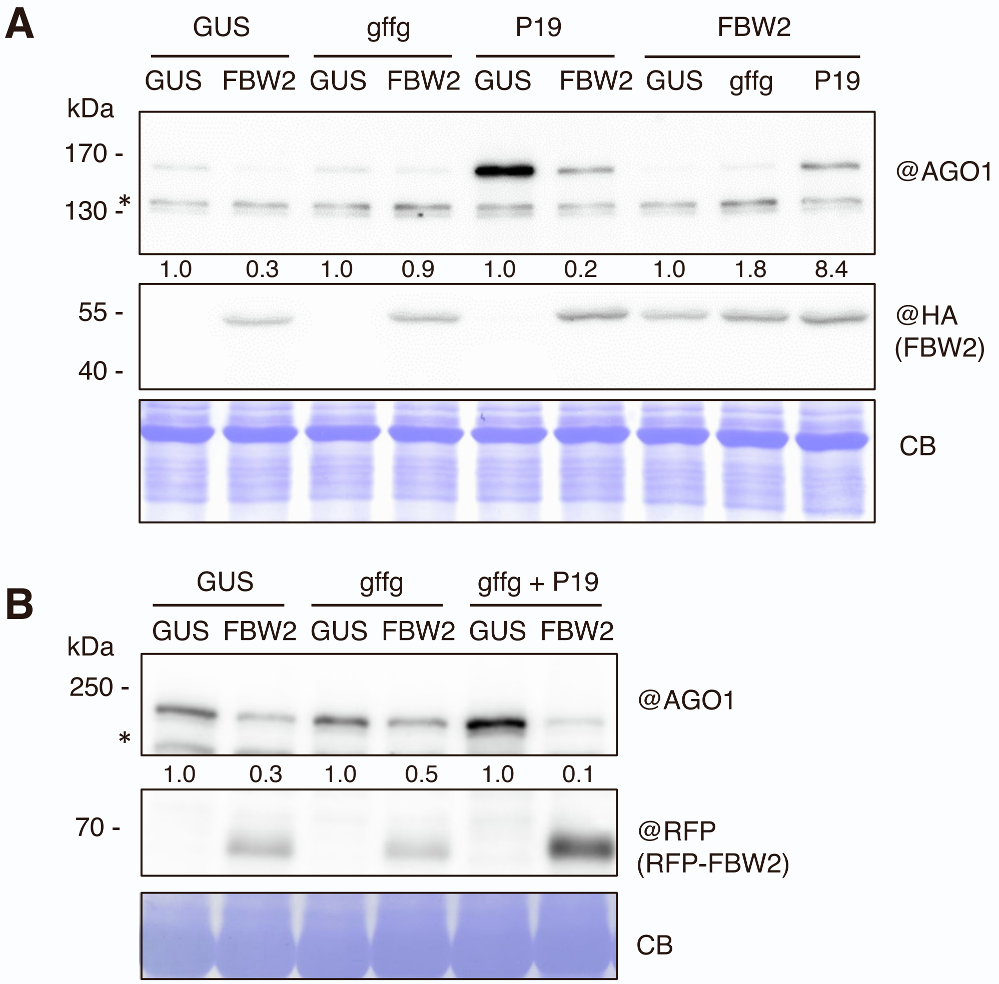

Figure S2. Effect of the inverted repeat *gffg* and P19 overexpression on AGO1 degradation by FBW2 (related to Figure 1).
(**A**) Western blot of protein extracts from 4 week-old *N. benthamiana* agroinfiltrated leaves with Agrobacteria harbouring the following binary vectors: 35S:CFP-AGO1, 35S:GUS (control), 35S:3HA-FWB2, 35S:gffg and 35S:P19 in combinations as specified. All constructs were infiltrated at 0,3 OD and tissues for protein analysis were sampled 3 days later. Coomassie blue (CB) staining was used as loading control. For this panel and also for panel B, AGO1 signal was quantified by ImageJ, normalized to the corresponding CB. Numbers are indicated below the panel as relative to the control set at 1.0. The "*" symbol indicates an aspecific cross-reacting band. The "@" symbol indicates hybridization with the corresponding antibodies.
(**B**) Western blot of protein extracts from 4 week-old *N. benthamiana* agroinfiltrated leaves with Agrobacteria harbouring the following binary vectors: 35S:RFP-AGO1, 35S:GUS (control), 35S:RFP-FWB2, 35S:gffg and 35S:P19 in combinations as specified. All constructs were infiltrated at 0,2 OD except for the experiment with P19 for which the infiltration solutions were adjusted to compensate the difference of expression (0.05 for AGO1, 0.1 for GUS and 0.1 for FBW2). Coomassie blue (CB) staining was used as loading control. The "*" symbol indicates an aspecific cross-reacting band. The "@" symbol indicates hybridization with the corresponding antibodies.

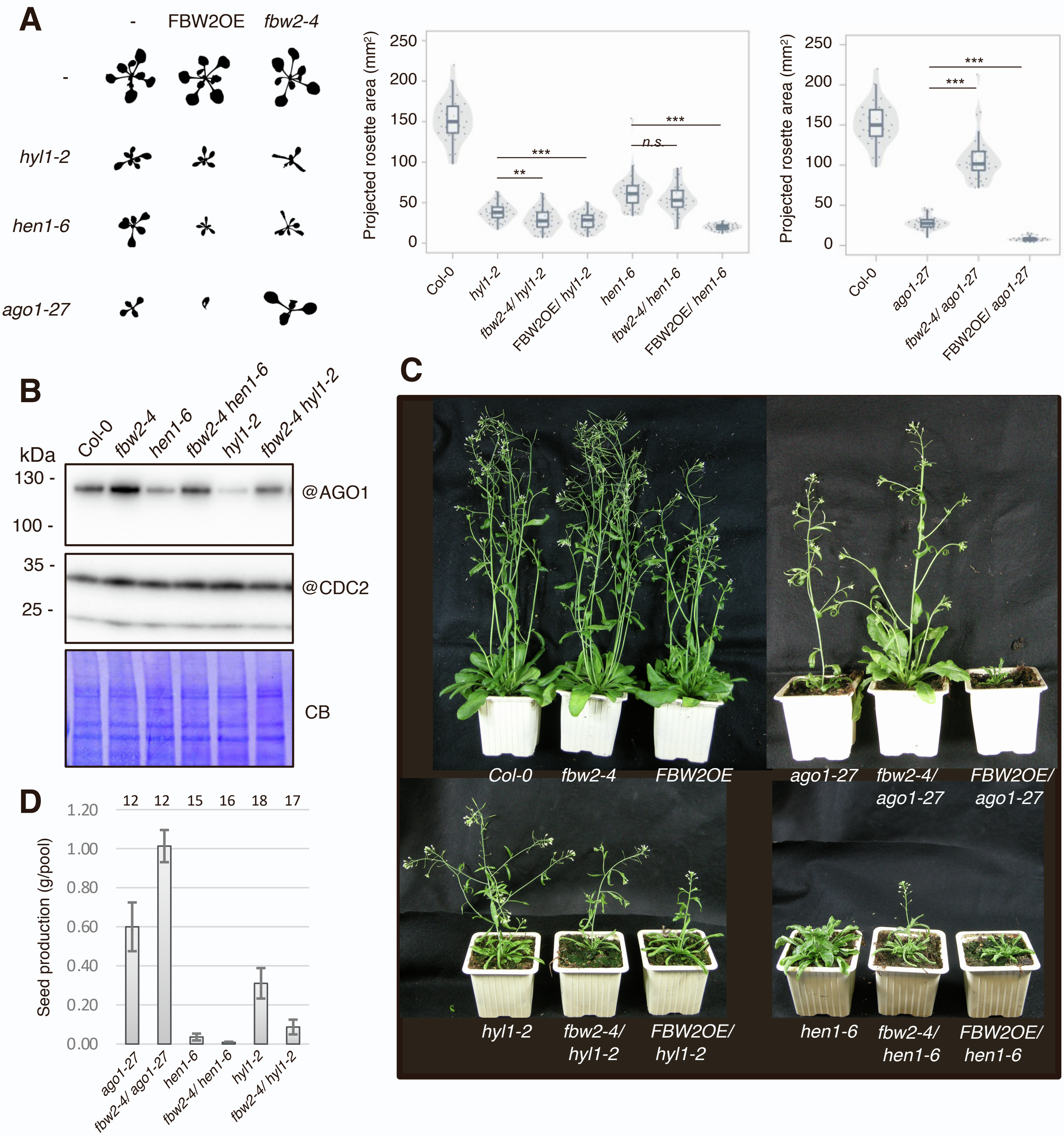

Figure S3. AGO1 protein level and phenotype of *hen1-6 fbw2-4* and *hyl1-2 fbw2-4* double mutants (related to Figure 4).
(**A**) Shape imprints and measurements of the rosette area of 17-days-old *in vitro* grown seedlings. Individual values are represented as dots within violin plots. Three independent biological replicates are shown, with n~12 plants per repeat. Statistical significance is shown only for the comparisons of the crosses with the single *hyl1-2, hen1-6* or *ago1-27* mutant. *** P < 0.001 (Students t-test), ** P < 0.01 (Students t-test), n.s. = not significant (P > 0.05, Students t-test).
(**B**) Western blot of protein extracts from 5 day-old seedlings of the indicated genotypes. Hybridization with the CDC2 antibody and Coomassie blue (CB) staining were used as loading controls.
(**C**) Pictures of three representative 40 days-old plants of Col-0, *hyl1-2*, *hen1-6*, *ago1-27* and their crosses with *fbw2-4* or 35S:3HA-FBW2 (FBW2OE) as indicated.
(D) Measurements of seed production (in gram). For each genotype, seeds from two individual plants were pooled and subsequently weighted. The number of pools measured per genotype is indicated on top of the graphs.

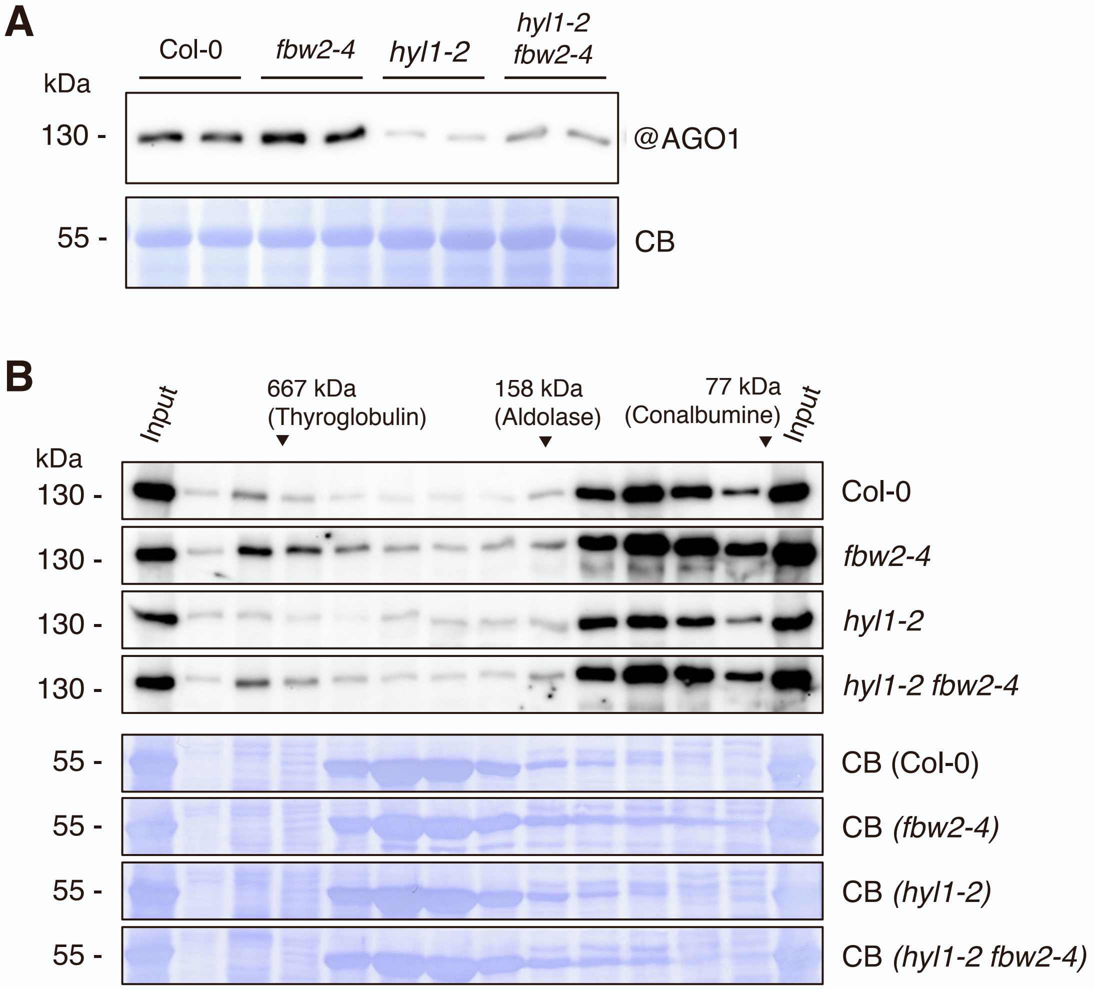

Figure S4. Loss of *FBW2* restores high molecular weight AGO1 complexes in *hyl1-2* (related to Figure 4).
(**A**) Western blot of protein extracts of 13 day-old seedlings of Col-0, *fbw2-4*, *hyl1-2*, and *hy1-2 fbw2-4*. Two biological replicates were made to illustrate the tendency of AGO1 homeostasis.
(**B**) Gel filtration analysis of AGO1-based RISC complexes from the same protein extracts shown in (A). Western blots of protein extracts from eluates of the gel filtration column (superdex 200 10/300 increase column on an AKTA Pure system) spanning from 6.75 to 13.75 ml elution volume. Molecular weights (kDa) of known protein sizes are indicated on top of the blot. "@" indicates hybridization with the corresponding antibodies. Coomassie blue staining was used as loading control.

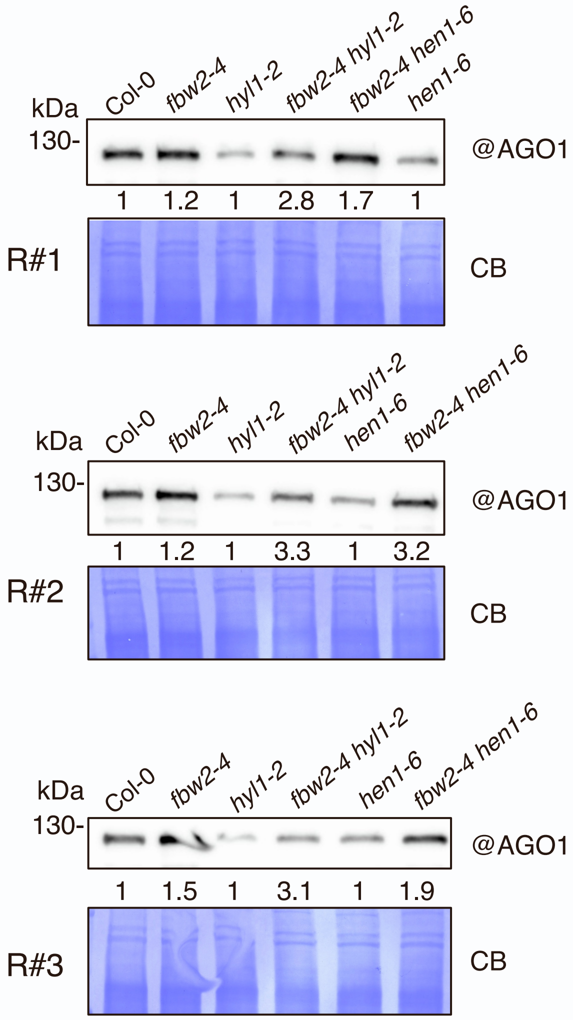

Figure S5. AGO1 protein level in different mutants used for RNA deep-sequencing experiments (related to Figure 5). Western blot of protein extracts from 8 day-old seedlings of the indicated genotypes. R#1, R#2, R#3 correspond to the three biological replicates. Hybridization with the @AGO1 antibody. Coomassie blue (CB) staining were used as loading controls. AGO1 signal was quantified by ImageJ, normalized to the corresponding Coomassie blue signal. Numbers are indicated below the panel as relative to Col-0, *hyl1-2 and hen1-6* set at 1.0.

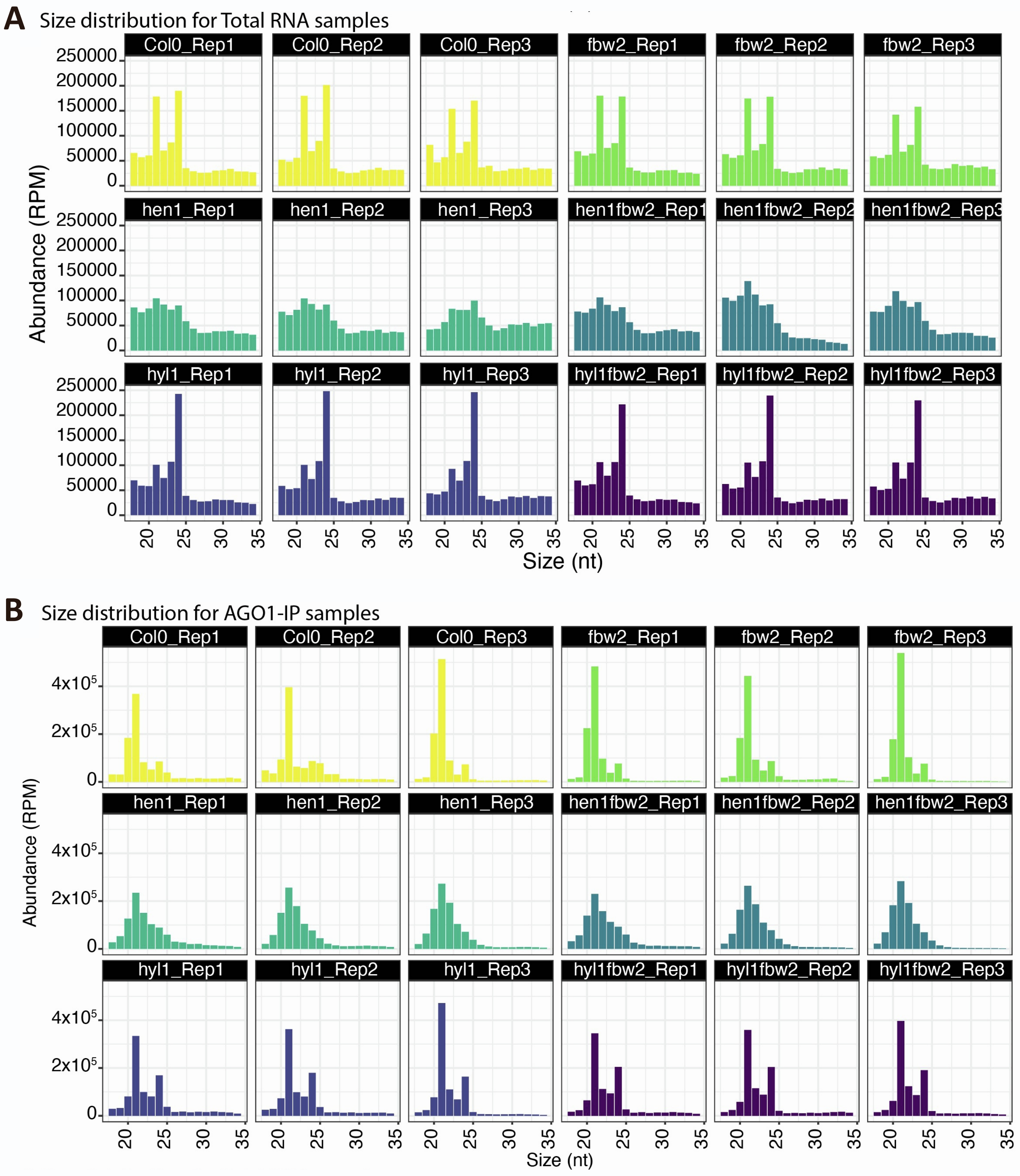

Figure S6. *FBW2* mutation has no effect in small RNA size distribution (related to Figure 5).
Size distribution of small RNA mapping to the Arabidopsis TAIR10 genome; the abundance of reads per million (RPM) of each size class was calculated for each source of data. The x axis indicates the small RNA size (from 18 to 34 nt) and the y axis indicates its abundance. Small RNA distribution for total RNA samples (A) and small RNA size distribution for AGO1 IP samples (B).

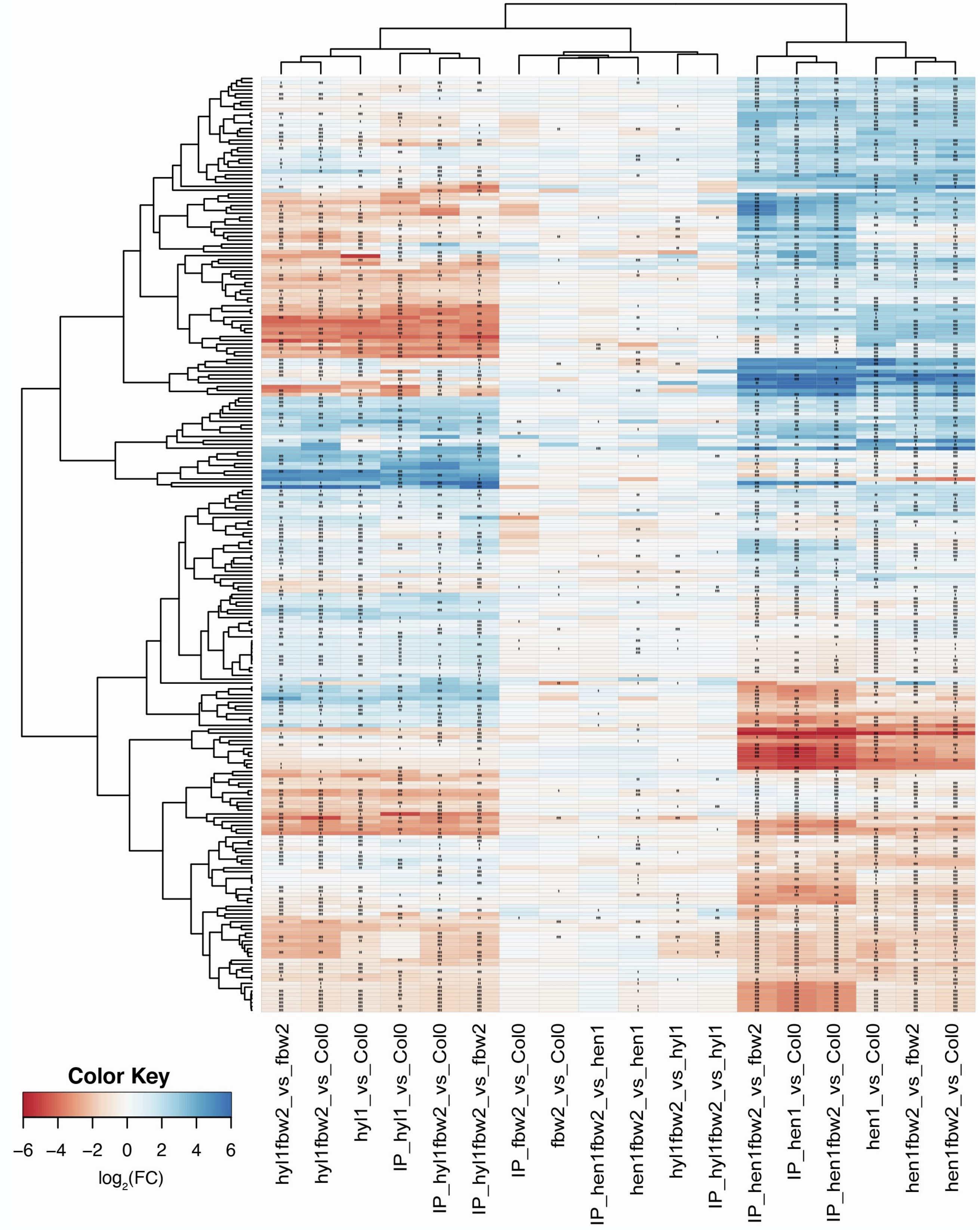

Figure S7. ***FBW2*** **mutation has a minimal effect in the miRNA population (related to Figure 5).**
Relative abundance of miRNA with significant differential expression in single and double mutants compared to wild-type (Col-0) and single mutants; the relative abundance is expressed as a heat map (see Key at the bottom), with the samples being compared indicated below each heat map (*Q value # 0.05, **Q value # 0.01 and **Q value #0.001).

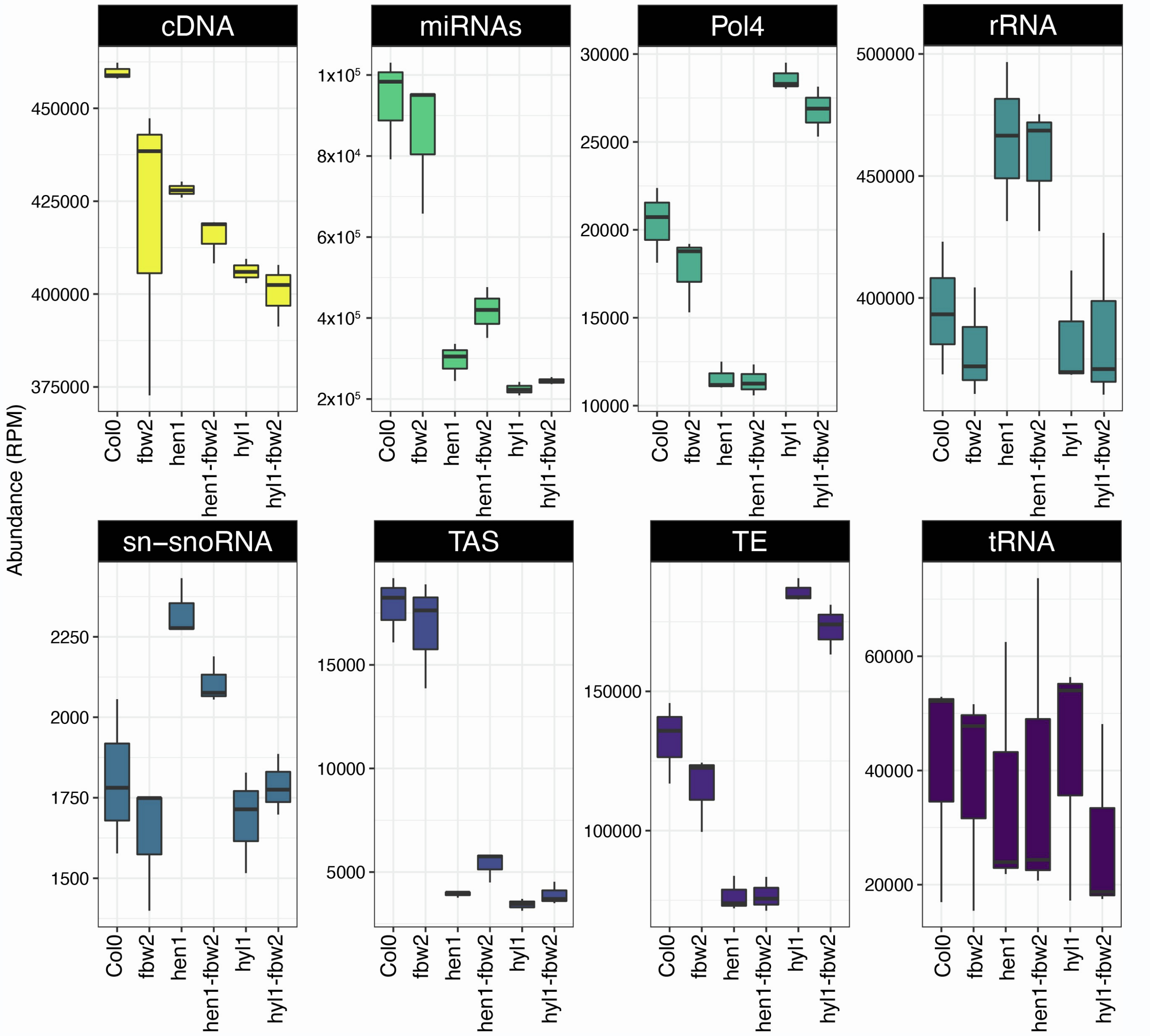

Figure S8. Pol4- and TE-derived sRNA reads are enriched in *hyl1 fbw2* double mutant, while no significant differences are observed in *fbw2* single mutants (related to Figure 5).
Boxplot representing the abundance of reads (in reads per million) mapping to eight different features of the Arabidopsis genome TAIR 10 for total RNA samples. These include the following from left to right: cDNA; mature miRNA; siRNA precursors dependent on Pol4; ribosomal RNA (rRNA); small nuclear and small nucleolar RNA (snRNA and snoRNA); TAS precursors; Transposable elements (TEs); tRNA-derived sRNA (tRNA); RPM, reads per million. Three biological replicates are included for each sample.

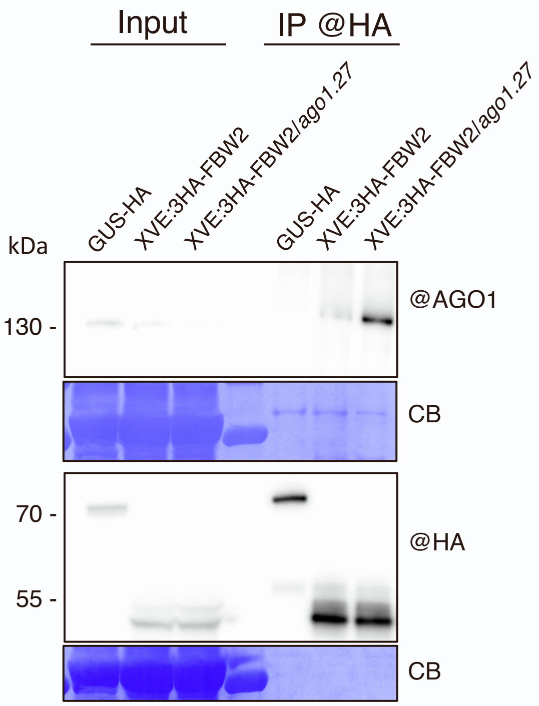

Figure S9. FBW2 interacts with AGO1 and exhibit a higher affinity toward the mutated form AGO1-27 (related to Figure 2 and 3).

Western blot of protein extracts from 7 day-old seedlings expressing XVE:3HA-FBW2 in Col-0 or *ago1-27* genetic backgrounds. 3HA-FBW2 was immunoprecipitated with anti-HA beads (μMACS HA Isolation Kit) after an induction of expression in liquid MS medium supplemented with β-Es (15μM) for 5 hours followed by 18 hours with by β-Es (15 μM) together with MLN4924 (25 μM). Protein extracts from 7 day-old seedlings expressing 35S-GUS-HA was used as a control for anti-HA immunoprecipitation. The "@" symbol indicates hybridization with the corresponding antibodies. Despite a lower accumulation level of AGO1-27 (input), the protein is more efficiently coprecipitated in FBW2 IPs than WT AGO1.
